# Neurexin-2: An inhibitory neurexin that restricts excitatory synapse formation in the hippocampus

**DOI:** 10.1126/sciadv.add8856

**Published:** 2023-01-06

**Authors:** Pei-Yi Lin, Lulu Y. Chen, Man Jiang, Justin H. Trotter, Erica Seigneur, Thomas C. Südhof

**Affiliations:** ^1^ Department of Molecular and Cellular Physiology, Stanford University School of Medicine, 265 Campus Dr., Stanford, CA 94305, USA.; ^2^ Howard Hughes Medical Institute, Stanford University School of Medicine, Stanford, CA 94305, USA.

## Abstract

Neurexins are widely thought to promote synapse formation and to organize synapse properties. Here we found that in contrast to neurexin-1 and neurexin-3, neurexin-2 unexpectedly restricts synapse formation. In the hippocampus, constitutive or neuron-specific deletions of neurexin-2 nearly doubled the strength of excitatory CA3➔CA1 region synaptic connections and markedly increased their release probability. No effect on inhibitory synapses was detected. Stochastic optical reconstruction microscopy (STORM) superresolution microscopy revealed that the neuron-specific neurexin-2 deletion elevated the density of excitatory CA1 region synapses nearly twofold. Moreover, hippocampal neurexin-2 deletions also increased synaptic connectivity in the CA1 region when induced in mature mice and impaired the cognitive flexibility of spatial memory. Thus, neurexin-2 controls the dynamics of hippocampal synaptic circuits by repressing synapse assembly throughout life, a restrictive function that markedly differs from that of neurexin-1 and neurexin-3 and of other synaptic adhesion molecules, suggesting that neurexins evolutionarily diverged into opposing pro- and antisynaptogenic organizers.

## Teaser

Neurexin-2 controls synaptic connectivity in hippocampal circuits by restricting excitatory connections throughout life.

## INTRODUCTION

In the brains of all animals, synapses connect neurons into neural circuits that process sensory inputs into motor outputs and that enable an animal to feel, plan, and remember. In a neural circuit, synapses not only transfer but also compute information, thus serving as fundamental information-processing units of the brain. Although the basic principles of synaptic transmission are well understood, less is known about the mechanisms guiding synapse assembly (*1*–*5*). Synapse formation and specification are regulated by transsynaptic adhesion molecules that transmit bidirectional signals, among which neurexins are arguably the best studied (*6*–*10*). In addition, secreted factors, including neurexin ligands such as cerebellins (*11*) and glial proteins such as glypicans (*12*), shape synapse organization. However, how synapse formation is controlled overall and how exuberant synapse formation induced by various synaptogenic molecules is restricted remains unclear.

In vertebrates, longer α-neurexins and shorter β-neurexins are encoded by three homologous genes (*Nrxn1*, *Nrxn2*, and *Nrxn3* in mice) that include separate promoters for α- and β-neurexin (*13*–*16*). In addition, the *Nrxn1* gene contains a third promoter driving transcription of even shorter γ-neurexins (*17*). Mutations in neurexin genes have been linked to multiple neuropsychiatric disorders. They are among the more common, albeit rare, genetic changes in schizophrenia, autism, and Tourette syndrome that affect thousands of patients (*18*–*23*). In human neurons, heterozygous deletions of *NRXN1*, either when engineered as conditional mutations or when resulting from a germline mutation in a patient, cause a robust decrease in excitatory synaptic strength (*24*, *25*), documenting the physiological impact of *NRXN1* mutations.

All neurexins are type I membrane proteins that exhibit the same overall domain structure (*6*, *10*). Extracellularly, α-neurexins contain six LNS (Laminin-G Neurexin Sex hormone-binding globulin) domains interspersed with three epidermal growth factor–like modules, while β-neurexins contain only a single LNS domain, namely the sixth LNS domain of α-neurexins. In both α- and β-neurexins, a highly glycosylated stalk sequence and a cysteine-loop domain separate their last (α-neurexins) or only LNS domain (β-neurexins) from their transmembrane region. Intracellularly, α- and β-neurexins feature a short cytoplasmic tail that binds to CASK (CAlcium calmodulin-dependent protein Serine Kinase) (*26*), which is also genetically linked to neurodevelopmental disorders (*27*). Neurexins are extensively alternatively spliced at six canonical positions that are present in all neurexins [except for splice site #6 (SS#6) that is absent from *Nrxn2*] and use highly similar sequences, generating thousands of isoforms (*15*, *28*, *29*). Moreover, in biochemical studies, all neurexins interact with the same array of ligands whose binding is often regulated by alternative splicing (*10*). For example, all α- and β-neurexins bind to cerebellins in a manner controlled by alternative splicing at SS#4 (*30*), whereas only α-neurexins, but not β-neurexins, bind to α-dystroglycan in a manner controlled by alternative splicing at SS#2 (*31*).

Initial studies of individual or combined deletions of *Nrxn1*α, *Nrxn2*α, and *Nrxn3*α in mice (with retained expression of β-neurexins) revealed that α-neurexins are essential for synaptic transmission and survival, and that their combined deletion causes a decrease in release probability but does not induce major changes in excitatory synapse numbers (*32*–*34*). These studies showed that the deletions of α-neurexins lower the activity of presynaptic voltage-gated Ca^2+^ channels, a finding that was extended in subsequent studies to deletions of all α- and β-neurexins (*35*–*37*). Individual deletions of all *Nrxn1* or *Nrxn3* isoforms also produced a decrease in synaptic strength in subsets of synapses (*24*, *25*, *38*–*40*), but *Nrxn2* deletions that affect all *Nrxn2* transcripts have not been examined. In general, the various neurexin deletions did not alter synapse numbers. In triple α-neurexin knockout (KO) mice, only inhibitory synapse numbers were decreased (*32*), while in triple α/β-neurexin KO mice excitatory climbing fiber and inhibitory parvalbumin-positive synapses in the cortex were partly lost (*35*). The view that emerged from these studies is that neurexins generally promote the functional, but not the physical, assembly of synapses by enabling the organization of the presynaptic release machinery and the postsynaptic receptor apparatus, but that neurexins do not have a central role in promoting the establishment of synapses.

The high degree of similarity among *Nrxn1*, *Nrxn2*, and *Nrxn3* suggested that all neurexins are functionally similar. However, recent studies revealed that, at least in some synapses, *Nrxn1, Nrxn2,* and *Nrxn3* perform distinct functions. Specifically, in subiculum synapses, alternative splicing of presynaptic *Nrxn1* at SS#4 regulates postsynaptic *N*-methyl-d-aspartate receptors (NMDARs), whereas the same alternative splicing of *Nrxn3* controls postsynaptic AMPA receptors (AMPARs) (*41*, *42*). These distinct functions of *Nrxn1* and *Nrxn3* are mediated via transsynaptic mechanisms that involve binding of presynaptic *Nrxn1* and *Nrxn3* to the same synaptic adaptor protein, cerebellin-2 (*43*). Cerebellin-2, in turn, forms a complex with postsynaptic GluD1 (for Glutamate D1 receptor), which is homologous to AMPARs and NMDARs and transmits the neurexin-cerebellin-binding signal (*43*). In the same synapses, alternative splicing of *Nrxn2* at SS#4 had no functional consequences and did not regulate either NMDARs or AMPARs (*42*).

These studies provided initial insights into the relative functions of *Nrxn1* and *Nrxn3*, but the relative overall roles of neurexins and the specific function of *Nrxn2* remained uncharacterized. What other function might neurexins mediate besides organizing the presynaptic active zone and regulating the postsynaptic receptor content? *Nrxn2*, in particular, is poorly studied, possibly because *Nrxn2*α KO mice, different from *Nrxn1*α and *Nrxn3*α KO mice, exhibited no survival phenotype, suggesting a less critical function (*32*). Thus, we generated *Nrxn2* conditional KO (cKO) mice that enable the deletion of all *Nrxn2* isoforms. Unexpectedly, we find that in the hippocampus, the loss of *Nrxn2* causes an increase both in the number of excitatory synapses and in the presynaptic release probability, suggesting a function in restricting synapse assembly. This *Nrxn2* function operates throughout life, demonstrating that *Nrxn2* has an opposing role to other neurexins: suppressing instead of promoting synapse assembly.

## RESULTS

### The *Nrxn2* deletion enhances synapse assembly

**Fig. 1.**
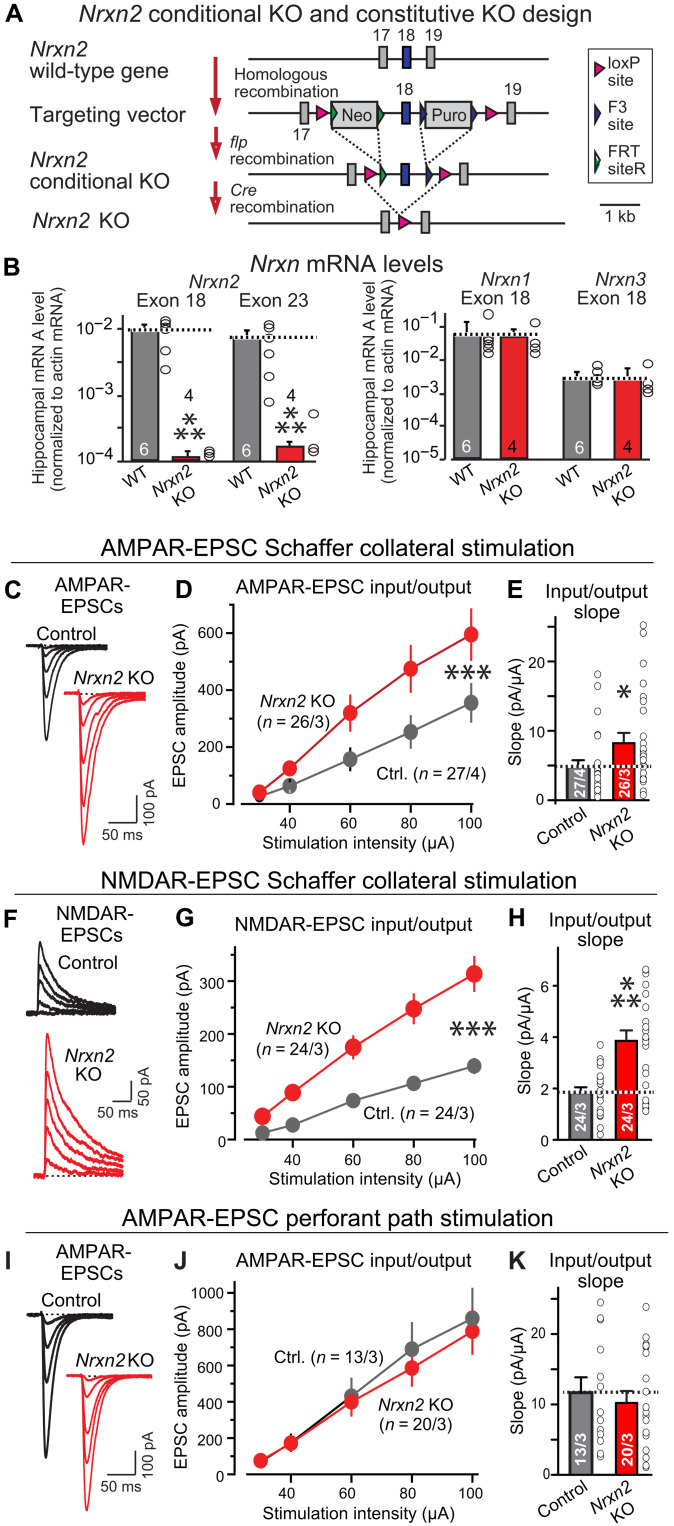
Constitutive deletion of *Nrxn2* increases CA3➔CA1 synaptic connections in the hippocampus. (**A**) *Nrxn2* cKO and constitutive KO strategy. Two selectable markers [puromycin (Puro) and neomycin (Neo)] were required to obtain embryonic stem cell clones with homologous recombination of the *Nrxn2* gene. Exon 18, the first exon shared between *Nrxn2*α and *Nrxn2*β, was flanked by loxP sites, enabling Cre-mediated deletion of both *Nrxn2*α and *Nrxn2*β. (**B**) *Nrxn2* cKO mice were bred with cytomegalovirus (CMV)–Cre mice to generate littermate wild-type (WT) and constitutive *Nrxn2* KO mice. The constitutive *Nrxn2* KO suppresses *Nrxn2* mRNA levels but leaves *Nrxn1* and *Nrxn3* mRNA levels unchanged. The exon 18 *Nrxn2* mRNA level measurements monitor the exon that is deleted, with the remaining 1% of mRNA detected likely because of background of quantitative reverse transcription polymerase chain reaction (RT-PCR) measurements. The decrease in the exon 23 mRNA levels is likely due to nonsense-mediated decay because the exon 18 deletion should not block *Nrxn2* transcription, only the production of a functional protein. (**C** to **E**) *Nrxn2* KO increases the AMPAR-mediated synaptic responses elicited by Schaffer collateral stimulation [(C) representative traces of AMPAR-EPSCs evoked by increasingly stronger stimuli and recorded at −70 mV in 50 μM picrotoxin and 50 μM D-D-AP-5 (2R)-amino-5-phosphonopentanoate) (2R)-amino-5-phosphonopentanoate); (D) input/output plot of the EPSC amplitude versus stimulus strength; (E) slope of the input/output relation]. (**F** to **H**) *Nrxn2* KO increases NMDAR-mediated synaptic responses elicited by Schaffer collateral stimulation [same as (C) to (E) except that the responses were recorded at a holding potential of +40 mV in the presence of 20 μM 6-cyano-7-nitroquinoxaline-2,3-dione (CNQX)]. (**I** to **K**) *Nrxn2* KO has no effect on AMPAR-mediated synaptic responses elicited by stimulation of entorhinal cortex–derived axons [same as (C) to (E)]. Data are means ± SEM; the numbers of neurons per mice analyzed are listed in bar graphs. Statistical assessments were performed by the Mann-Whitney test comparing KO to control (B, E, H, and K) or by two-way analysis of variance (ANOVA) tests (D, G, and J), with *P < 0.05, **P < 0.01, and ***P < 0.001. Ctrl., control.

To examine the function of *Nrxn2*, we generated cKO and constitutive KO mice that delete expression of all functional *Nrxn2* transcripts (Fig. 1A), in contrast to the constitutive *Nrxn*2α KO mice that we reported earlier, and that do not delete *Nrxn*2β expression (*32*). Note that we had previously generated another line of *Nrxn2*α/β mutant mice that, unfortunately, included a gene rearrangement and did not actually delete *Nrxn2* but that were characterized by others (*44*). Constitutive *Nrxn2* KO mice were viable and fertile and exhibited decreased levels of *Nrxn2* mRNAs (presumably because of nonsense-mediated decay) without a change in the levels of *Nrxn1* and *Nrxn3* mRNAs (Fig. 1B). We examined hippocampal cryosections from constitutive *Nrxn2* KO mice by immunofluorescence for synaptic markers but found no major changes in the overall organization of the hippocampus (fig. S1, A and B).

The constitutive *Nrxn2* deletion enabled us to examine the effect of a chronic *Nrxn2* ablation in mice on the expression of other neurexins and synaptic marker proteins. We first measured neurexin protein levels in *Nrxn2* KO mice using quantitative immunoblotting with seven different antibodies against neurexins. These antibodies were raised against different neurexin sequences that are similar in all three neurexins and were validated, in part, using neurexin-triple KO cultures (*45*, *46*). We used so many different antibodies because antibodies to neurexins have traditionally been difficult to raise and because high-affinity isoform-specific antibodies to neurexins are not widely available, making it difficult to measure the levels of individual neurexins. We detected no changes in the overall levels of neurexins in *Nrxn2* KO hippocampus compared to wild-type (WT) controls with any of the antibodies (fig. S1, C and D). Thus, the *Nrxn2* deletion does not greatly alter the levels of *Nrxn1* and *Nrxn3* proteins, consistent with the lack of a change in *Nrxn1* and *Nrxn3* mRNA levels upon deletion of *Nrxn2*. In these experiments, total neurexin levels did not decrease after the *Nrxn2* deletion. This is probably because *Nrxn2* exhibits the lowest expression levels among neurexin isoforms (*28*) and, therefore, its loss does not cause a major change in total neurexin protein levels. Moreover, the neurexin antibodies raised to Nrxn1 and Nrxn3 sequences may not react as strongly with *Nrxn2* as with *Nrxn1* or *Nrxn3*.

In addition to neurexins, we analyzed the levels of neuroligins and CASK that interact with neurexins, as well as those of various synaptic marker proteins (fig. S1, E and F). Again, we detected no significant changes in any of these proteins. Thus, constitutive *Nrxn2* KO mice do not experience an up-regulation of *Nrxn1* and/or *Nrxn3* expression, a major restructuring of their synaptic proteome, or a large change in their cytoarchitecture.

To identify functional changes induced by the constitutive *Nrxn2* deletion, we monitored Schaffer collateral CA3➔CA1 or entorhinal cortex➔CA1 synaptic transmission in acute hippocampal slices. We used input/output measurements with extracellular stimulations to quantify AMPAR- and NMDAR-mediated synaptic responses independent of electrode placements (Fig. 1, C to H). Unexpectedly, the *Nrxn2* deletion massively elevated both AMPAR-mediated excitatory postsynaptic currents (AMPAR-EPSCs) (~80% increase) (Fig. 1, C to E) and NMDAR-mediated EPSCs (NMDAR-EPSCs) (~120% increase) elicited by Schaffer collateral stimulation (Fig. 1, F to H). The constitutive *Nrxn2* deletion, however, had no effect on AMPAR-EPSCs elicited by entorhinal cortex afferent stimulation (Fig. 1, I to K). No changes in EPSC kinetics, neuronal input resistance, or capacitance were observed (fig. S2).

### Generation of neuron-specific *Nrxn2* deletion mice

**Fig. 2.**
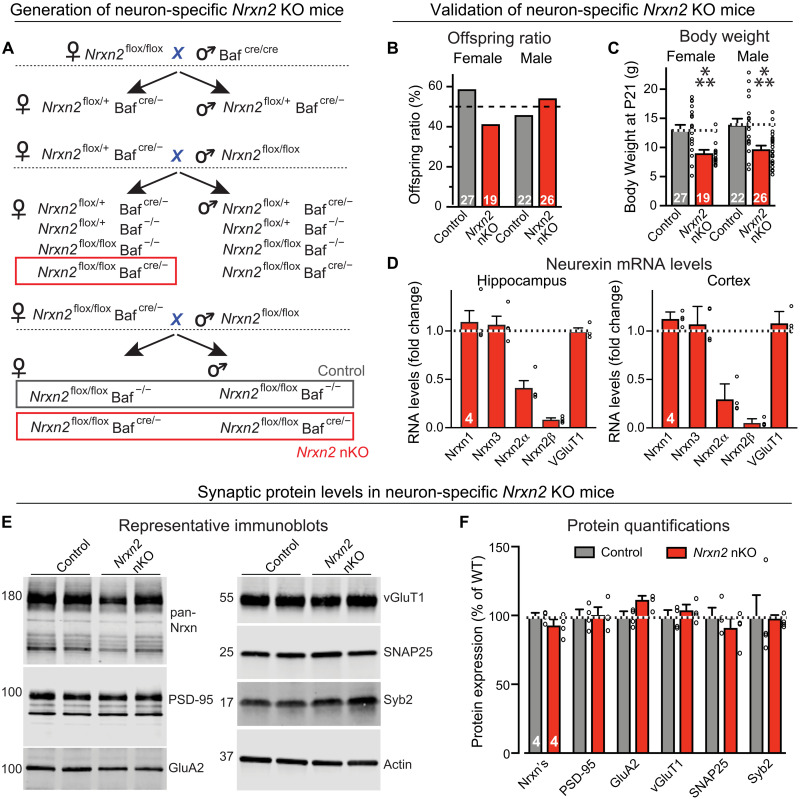
The pan-neuronal deletion of *Nrxn2* (*Nrxn2* nKO) partly decreases *Nrxn2* mRNAs but does not affect the levels of other mRNAs or protein levels. (**A**) Breeding strategy for generating pan-neuronal *Nrxn2* KO (*Nrxn2* nKO) mice by crossing *Nrxn2* cKO mice with Baf53b-Cre mice (*47*). (**B**) The *Nrxn2* nKO does not impair mouse survival. The graph depicts the genotype distribution in surviving offspring from matings of homozygous *Nrxn2* cKO (control) and *Nrxn2* nKO mice, with an expected 50% offspring survival ratio for *Nrxn2* cKO control and *Nrxn2* nKO mice, assessed at postnatal day 21 (P21). (**C**) Body weight of the mice analyzed in (B). (**D**) Quantification of the indicated neurexin mRNA levels in the hippocampus and cortex of littermate *Nrxn2* cKO control and *Nrxn2* nKO mouse brains, expressed as the fraction of the mRNA levels in the nKO mice compared to controls. Note that the remaining *Nrxn2*α mRNA levels are higher than *Nrxn2*β mRNA levels because *Nrxn2*α but not *Nrxn2*β is also expressed in astrocytes and OPCs (oligodendrocyte precursor cells). (**E** and **F**) Immunoblotting analyses show that the neuron-specific *Nrxn2* deletion (*Nrxn2* nKO) does not significantly alter the levels of key synaptic proteins, including that of total neurexins [(E) representative blots; (F) summary graph of protein levels as determined by quantitative blotting using fluorescent secondary antibodies and Licor detection]. Data in (B) to (D) and (F) are means ± SEM; the numbers of analyzed mice or of cells per mice are shown in the bars. Statistical analyses were performed using the Mann-Whitney test comparing KO to WT, with ***P < 0.001.

Single-cell RNA sequencing (RNA-seq) studies show that *Nrxn2* is expressed not only in neurons but also in glia (fig. S3). Depending on how various laboratories processed primary RNA-seq data, the relative *Nrxn2* expression levels differed between different types of glia and neurons, but in all studies the *Nrxn2* levels in astrocytes and oligodendrocyte precursor cells were similar to those of neurons (fig. S3). It is thus unclear whether the restriction of synaptic connectivity by *Nrxn2* that emerges from our analysis of constitutive KO mice is due to a deletion of *Nrxn2* in neurons, glia, or both. To dissect this issue, we crossed the *Nrxn2* cKO mice with Baf53b-Cre mice that express Cre recombinase (Cre) selectively in neurons but not glia (*47*). The resulting neuron-specific *Nrxn2* KO mice (referred to as “*Nrxn2* nKO” mice) were viable and fertile (Fig. 2, A and B) but exhibited a decrease in body weight (Fig. 2C). Analyses of mRNA levels revealed that the neuron-specific *Nrxn2* deletion caused a 60 to 70% decrease in *Nrxn2*α and >90% decrease in *Nrxn2*β mRNA levels in the brain (Fig. 2D). This result is consistent with the observation that β-neurexin isoforms are more neuron-specific than α-neurexin isoforms (*40*), and that, the single-cell RNA-seq studies notwithstanding, the majority of *Nrxn2* mRNA is expressed by neurons. Alternatively, it is possible that the Baf53b-Cre also induces nonneuronal recombination, but this seems highly unlikely given extensive data demonstrating neuron specificity of this Cre line (*47*). Quantitative immunoblotting revealed that the overall levels of neurexins were unchanged *Nrxn2* nKO mice similar to the constitutive *Nrxn2* KO mice, as were the levels of selected synaptic markers (Fig. 2, E and F).

### The neuron-specific *Nrxn2* deletion increases CA3➔CA1 synaptic transmission, at least in part, by elevating the presynaptic release probability

**Fig. 3.**
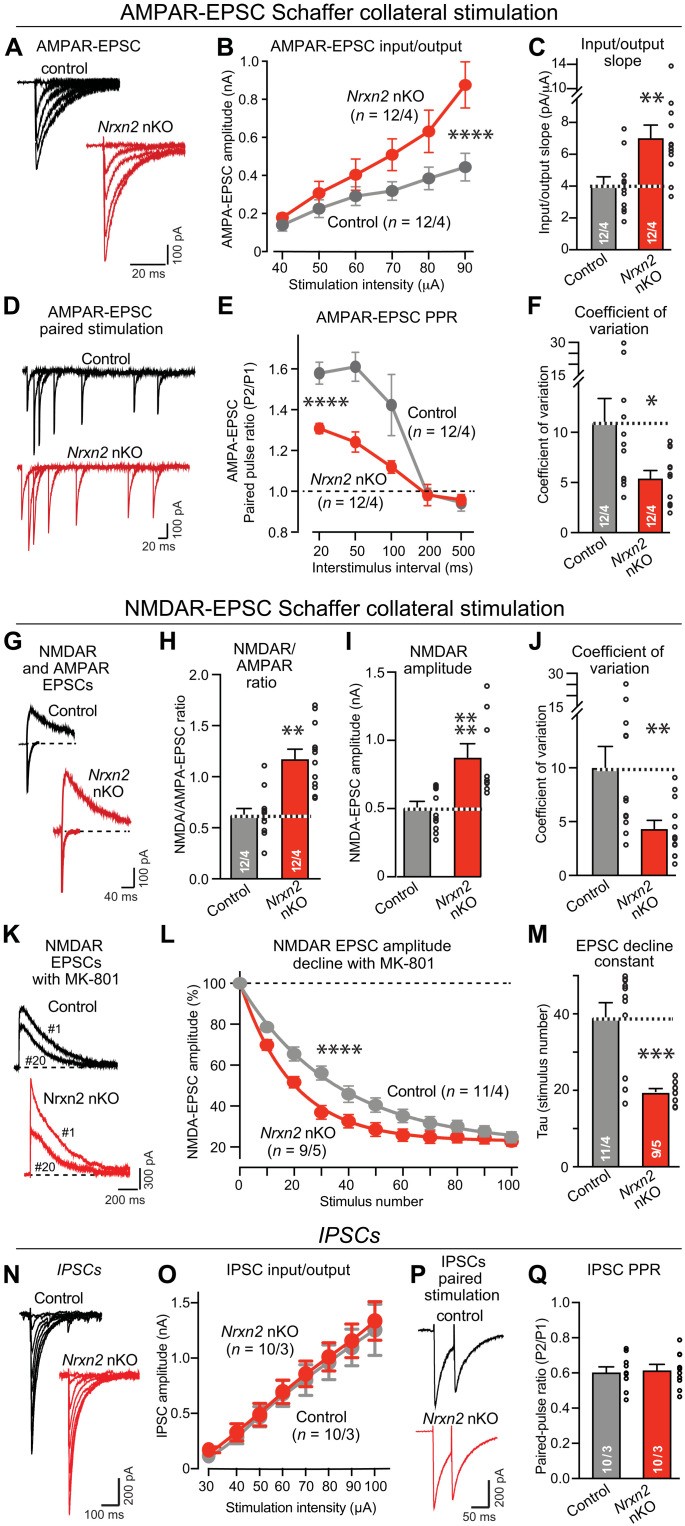
The pan-neuronal deletion of *Nrxn2* (*Nrxn2* nKO) elevates CA3➔CA1 synaptic connectivity and increases the release probability at CA3➔CA1 synapses. (**A** to **C**) *Nrxn2* neuron-specific KO (nKO) increases AMPAR-mediated synaptic responses elicited by Schaffer collateral stimulation in acute slice from littermate control and *Nrxn2* nKO mice [(A) representative traces of AMPAR-EPSCs evoked by electrical stimulation with increasing intensity; (B) input/output curve; (C) summary graph of the input/output slope]. (**D** to **F**) *Nrxn2* nKO increases presynaptic release probability as demonstrated by a decreased paired-pulse ratio (PPR) and a lower coefficient of variation of AMPAR-EPSCs [(D) representative traces; (E) summary plot of PPRs; (F) summary graph of the coefficient of variation]. (**G** to **J**) *Nrxn2* nKO enhances NMDAR/AMPAR ratio by increasing NMDAR-mediated synaptic responses more strongly than AMPAR-mediated responses [(G) representative traces of NMDAR-EPSCs and AMPAR-EPSCs monitored in the same cell at a +40- and −70-mV holding potential; (H to J) summary graphs of the NMDAR-EPSC/AMPAR-EPSC ratio (H), the absolute NMDAR-EPSC amplitude (I), and the coefficient of variation of NMDAR-EPSCs (J)]. (**K** to **M**) *Nrxn2* nKO increases presynaptic release probability as measuring the rate of NMDAR-EPSC decline during 0.1-Hz stimulus trains in the presence of 20 μM MK-801 [(K) representative traces of the 1st and 20th NMDAR-EPSCs in the train; (L) normalized NMDAR-EPSC amplitudes in the presence of MK-801; (M) summary graph of the decay constant]. (**N** and **O**) The *Nrxn2* nKO has no effect on inhibitory postsynaptic currents (IPSCs) [(N) representative traces of IPSCs evoked by electrical stimulation with increasing intensity; (O) input/output curve]. (**P** and **Q**) *Nrxn2* nKO has no effect on PPR in inhibitory synapses monitored with a 50-ms interstimulus interval [(P) representative traces; (Q) summary graph of the PPR). Numerical data are means ± SEM; the numbers of analyzed cells per mice are listed in bar graphs. Statistical assessments were performed by two-way ANOVA (B, E, L, and O) or Mann-Whitney tests comparing the *Nrxn2* nKO to controls, with *P < 0.05; **P < 0.01, ***P < 0.001, and ****P < 0.0001.

We examined synaptic transmission in *Nrxn2* nKO mice using electrophysiological measurements in acute hippocampal slices from young adult mice [postnatal day 35 (P35) to P45]. Input/output measurements of AMPAR-mediated Schaffer collateral EPSCs identified a large increase (~80%) in excitatory synaptic strength (Fig. 3, A to C), which mirrors the increase observed in constitutive *Nrxn2* KO mice (Fig. 1). Paired-pulse measurements of AMPAR-EPSCs revealed a decrease in paired-pulse facilitation suggestive of an increase in release probability (Fig. 3, D and E). Moreover, the coefficient of variation of AMPAR-EPSCs was significantly lower, consistent with an increase in release probability (Fig. 3F). We also measured the ratio of NMDAR-EPSCs/AMPAR-EPSCs and the amplitude of NMDAR-EPSCs and observed an increase in both (Fig. 3, G to I). In addition, the coefficient of variation of NMDAR-EPSCs was decreased by more than 50% (Fig. 3J). In these measurements, no significant difference between male and female mice was detected (fig. S4).

To rigorously test whether the release probability was increased, we directly measured the release probability. We monitored the rate of decline in synaptic NMDAR-EPSCs induced in the presence of MK-801, an NMDAR antagonist that only blocks activated NMDARs. Because of this property, the rate of NMDAR-EPSC decline in the presence of MK-801 is inversely proportional to the release probability (*48*, *49*). Notably, the neuronal *Nrxn2* deletion caused a twofold acceleration of the rate of the MK-801–induced decline in NMDAR-EPSCs, confirming that it increases the release probability (Fig. 3, K to M). Parallel measurements of inhibitory postsynaptic currents (IPSCs) did not uncover any change in the synaptic strength or the presynaptic release probability of inhibitory synapses (Fig. 3, N to Q).

### Neuron-specific *Nrxn2* deletion also robustly enhances the number of CA3➔CA1 synapses

**Fig. 4.**
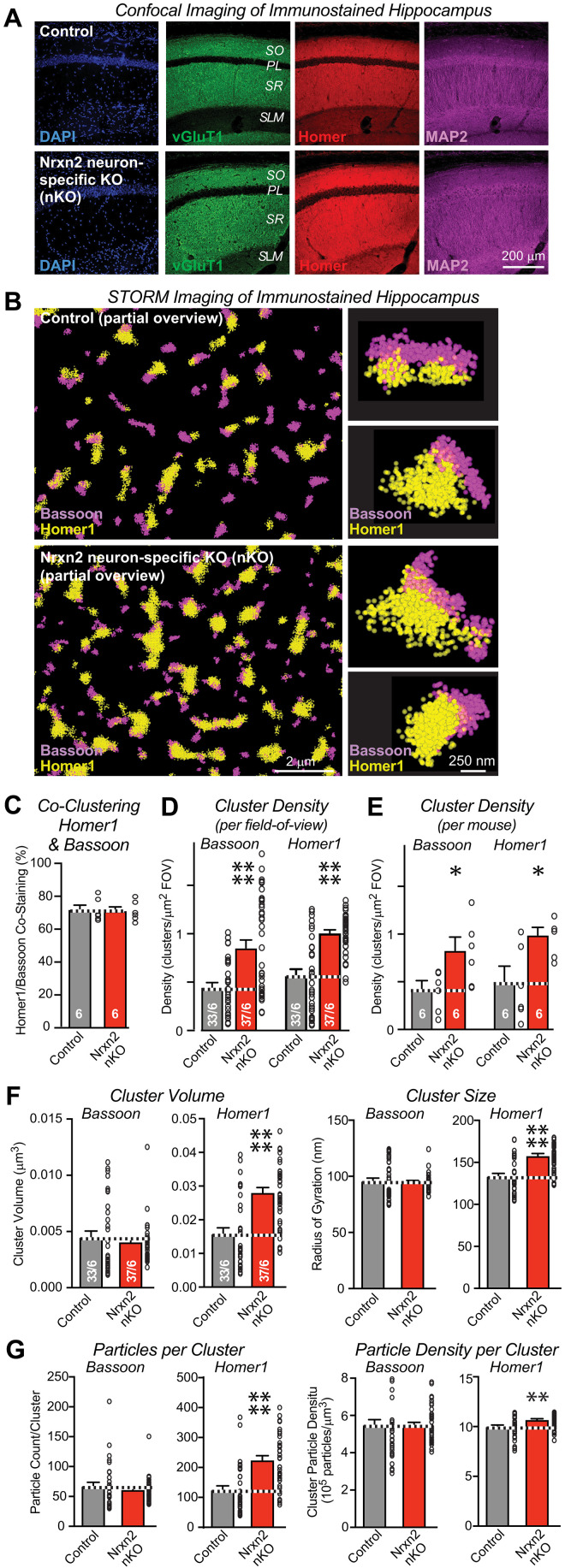
STORM superresolution microscopy reveals that the pan-neuronal *Nrxn2* deletion (*Nrxn2* nKO) increases the excitatory synapse density in the CA1 region. (**A**) The pan-neuronal deletion of *Nrxn2* does not alter the overall synaptic architecture of the hippocampal CA1 region. Representative images show low-magnification confocal views of cryosections that were labeled for 4′,6-diamidino-2-phenylindole (DAPI) (blue), vGluT1 (green), Homer1 (red), and MAP2 (magenta). (**B**) Representative dSTORM images of presynaptic Bassoon clusters (magenta) and postsynaptic Homer1 clusters (yellow) in *S. radiatum* of the CA1 region from control and *Nrxn2* nKO mice. Note that the expanded images on the right we taken from random low-magnification images of synapses and are not necessarily present in the partial overview on the left, but all images are publicly available at https://purl.stanford.edu/nb252dn4150. (**C**) Summary graph documenting that the majority of Bassoon and Homer1 clusters colocalize and that their colocalization is unaffected by the neuron-specific *Nrxn2* deletion. (**D** and **E**) Summary graphs showing that the *Nrxn2* nKO markedly increases the density of Bassoon and Homer1 clusters in the *S. radiatum* of the CA1 region, as analyzed by dSTORM quantifying the number of clusters either per field of view (FOV) (D) or per mouse (E). (**F** and **G**) Summary graphs demonstrating that the pan-neuronal deletion of *Nrxn2* increases the volume [(F) left], size [(F) right], particle numbers [(G) left], and particle density [(G) right] of Homer1 clusters but has no detectable effect on these parameters in Bassoon clusters. Data in (C) to (F) are means ± SEM; the numbers of analyzed mice (C and E) or sections per mice (D and F) are listed in bar graphs. Statistical assessments were performed by Mann-Whitney tests comparing the nKO to controls, with *P < 0.05, **P < 0.01, and ****P < 0.0001.

We next analyzed the excitatory synapse density in the CA1 region of the hippocampus from *Nrxn2* nKO mice by immunofluorescence. Confocal overviews again failed to detect major changes in the overall hippocampal architecture (Fig. 4A), and quantifications of CA1 region sections stained for presynaptic marker vGluT1 (vesicular Glutamate Transporter 1), postsynaptic marker Homer, and MAP2 (Microtubule-Associated Protein 2) showed that the overall immunofluorescence signal was not changed by the *Nrxn2* nKO (fig. S5). Because the high density of synapses in the CA1 region renders accurate synapse quantifications by confocal microscopy difficult, we used direct stochastic optical reconstruction microscopy (dSTORM). dSTORM imaging of hippocampal sections that were double-labeled for the presynaptic marker Bassoon and the postsynaptic marker Homer1 revealed that, as expected, Bassoon and Homer1 were localized adjacent to each other in large “macroclusters” (Fig. 4, B and C) (*45*, *50*). Notably, the density of Bassoon and Homer1 macroclusters was greatly elevated (>100% increase) in *Nrxn2* nKO mice (Fig. 4, D and E). We quantified this increase by two approaches: first, as cluster numbers per field of view, a standard procedure that enhances statistical significance because it is based on pseudo-replicates (Fig. 4D); second, as cluster numbers per mouse as true replicates (Fig. 4E). Both procedures uncovered a similarly large increase in synapse density in *Nrxn2* nKO mice, confirming the overall conclusion. In analyzing the dSTORM data, we also noted that the *Nrxn2* nKO enlarged the size of Homer1 but not of Bassoon clusters, as revealed by an increase (~75%) in cluster volume, cluster size, particle numbers per cluster, and particle density per cluster (Fig. 4, F and G). This finding suggests that the *Nrxn2* nKO enhances the size of postsynaptic but not presynaptic specializations in addition to elevating the synapse density. Viewed together, the results of the STORM imaging experiments and electrophysiological recordings from *Nrxn2* nKO mice indicate that the neuronal deletion of *Nrxn2* causes a large increase in excitatory synaptic connectivity due to both an increase in synapse density and in release probability.

### The conditional *Nrxn2* deletion in adult mice enhances synaptic connectivity

**Fig. 5.**
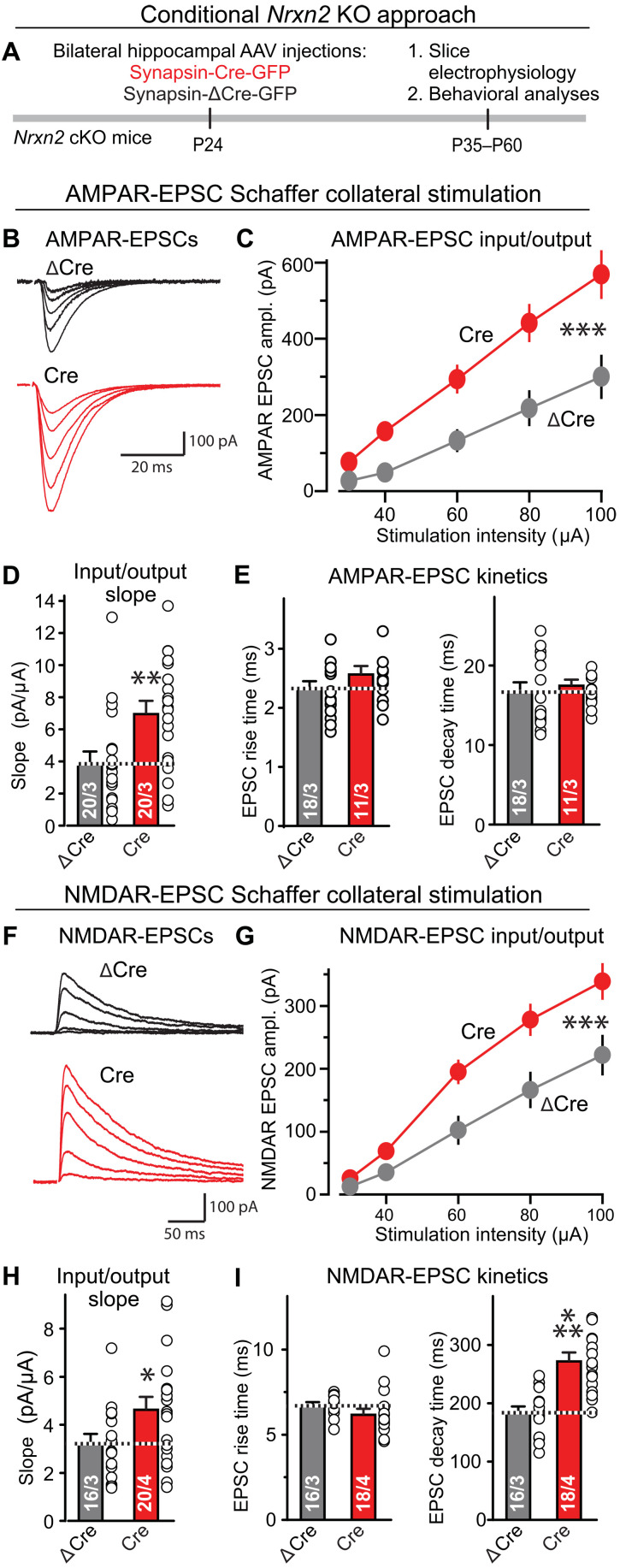
Conditional deletion of *Nrxn2* from the hippocampus of adolescent mice enhances the strength of CA3➔CA1 synaptic connections. (**A**) Experimental approach. The hippocampal formation of *Nrxn2* cKO mice was stereotactically infected at P24 with AAVs expressing inactive (ΔCre, control) or active Cre recombinase (Cre), and mice were analyzed by electrophysiologically and behaviorally 2 to 5 weeks later. (**B** to **E**) Conditional *Nrxn2* deletion at P24 increases AMPAR-EPSCs elicited by Schaffer collateral stimulation [(B) representative traces of AMPAR-EPSCs induced by increasingly stronger electrical stimulation and recorded at −70 mV in 50 μM picrotoxin and 50 μM D-AP-5 (2R)-amino-5-phosphonopentanoate); (C) input/output plot of the EPSC amplitude versus stimulus strength; (D) slope of the input/output relation; (E) rise and decay times of AMPAR-EPSCs]. (**F** to **I**) Conditional *Nrxn2* deletion also increases NMDAR-mediated synaptic responses elicited by Schaffer collateral stimulation. Same as (B) to (E), except those responses were recorded at a holding potential of +40 mV in the presence of 20 μM CNQX instead of 50 μM D-AP-5 (2R)-amino-5-phosphonopentanoate). Data are means ± SEM; the numbers of neurons per mice analyzed are listed in the graphs. Statistical assessments were performed by two-way ANOVA (C and G) or Mann-Whitney test (all bar graphs) comparing the Cre condition to the ΔCre controls, with *P < 0.05, **P < 0.01, and ***P < 0.001.

Does *Nrxn2* restrict developmental synapse formation, or does it control synapse numbers throughout life? To address this question, we investigated whether a postdevelopmental conditional deletion of *Nrxn2* alters synapse assembly. We stereotactically infected the hippocampal formation of *Nrxn2* cKO mice with AAVs (Adeno-Associated Viruses) encoding enhanced green fluorescent protein (EGFP)–tagged inactive mutant (ΔCre; used as a control) or active Cre at P24. At P35 to P45, we monitored the strength of AMPAR- and NMDAR-mediated EPSCs by measuring input/output curves in acute slices (Fig. 5A). We observed a significant increase in evoked AMPAR-EPSCs (~80%) (Fig. 5, B to E) and NMDAR-EPSCs (~40%) (Fig. 5, F to I). The EPSC kinetics were largely unchanged except for an increase in NMDAR-EPSC decay times.

The unexpected increase in synapse assembly induced by the *Nrxn2* deletion raises the question whether this phenotype is truly due to a presynaptic function of *Nrxn2* similar to that of other neurexins (*32*, *34*, *39*) or whether it might reflect a previously unidentified postsynaptic role of *Nrxn2*. To address this question, we examined the effect of selective postsynaptic conditional *Nrxn2* deletions in the hippocampus. No effect of these deletions on synaptic inputs onto a postsynaptic neuron was detected, suggesting that, consistent with previous studies on neurexins (*32*, *39*), *Nrxn2* acts selectively presynaptically (fig. S6).

**Fig. 6.**
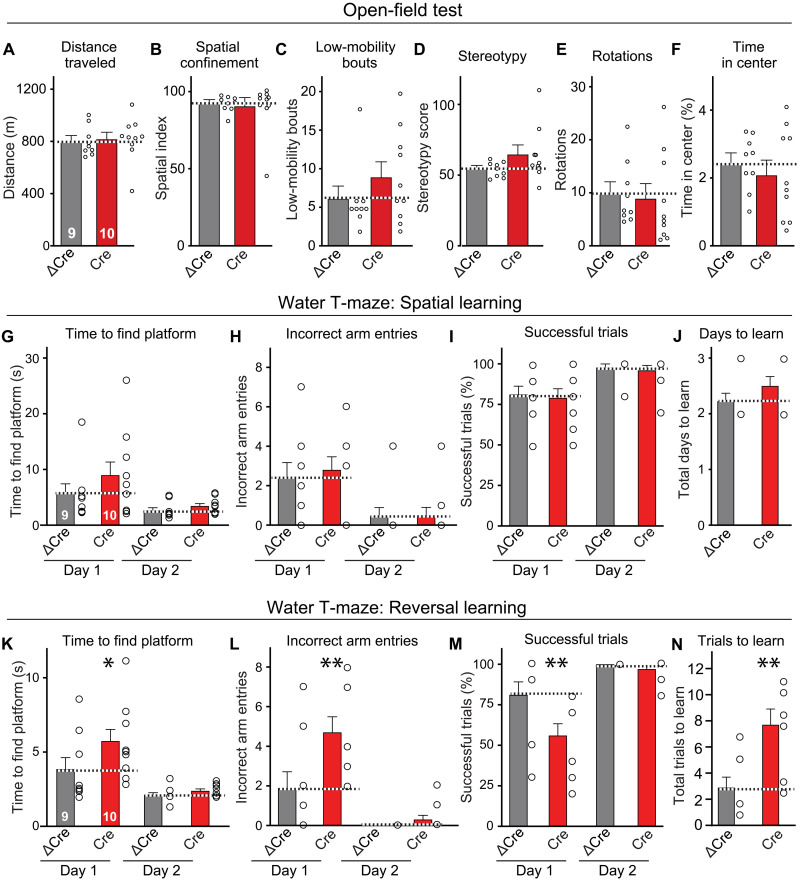
Conditional deletion of *Nrxn2* in the hippocampus of adolescent mice selectively impairs spatial reversal learning. (**A** to **F**) The conditional *Nrxn2* deletion in the hippocampal formation at P24 has no effect on the mouse behavior in the open-field test, arguing against a global disruption in brain function. (**G** to **J**) Behavioral assays show that the conditional deletion of *Nrxn2* in the hippocampus does not impair acquisition of spatial memory in the water T-maze test. For more assays, see fig. S7. (**K** to **N**) Conditional deletion of *Nrxn2* in the hippocampus induces a significant impairment in spatial reversal learning in the water T-maze test, suggesting an inability in relearning a new spatial situation. Data are means ± SEM; the numbers of mice analyzed are listed in the graphs. Statistical assessments were performed by two-way ANOVA (G to I and K to M) or Mann-Whitney test (A to F, J, and N) comparing the Cre condition to the ΔCre controls, with *P < 0.05 and **P < 0.01

In a final set of experiments, we explored the potential behavioral effect of increasing synaptic connections in the hippocampus and analyzed *Nrxn2* cKO mice that were infected with AAVs at P24. We observed no major changes in *Nrxn2* cKO mice in the open-field test, fear conditioning training or recall, or passive avoidance tests and detected only a small impairment in the rotarod test (Fig. 6, A to F, and fig. S7). However, when we tested the *Nrxn2* cKO mice in the water T-maze, they learned normally but exhibited a severe deficit in reversal learning (Fig. 6, G to N). Mice with hippocampal inactivation of *Nrxn2* were slower in learning the new platform position, suggesting that they were less able to adjust their behavior to a new spatial context and thus exhibited diminished cognitive flexibility.

## DISCUSSION

Here we show that *Nrxn2*, different from *Nrxn1* and *Nrxn3*, functions to restrict, instead of enabling, synapse assembly in the hippocampus. This finding was confirmed by electrophysiological analyses of three different genetic *Nrxn2* manipulations, constitutive global deletions (Fig. 1), neuron-specific deletions (Fig. 3), and conditional deletions in which *Nrxn2* was selectively ablated postdevelopmentally (Fig. 5). The presence of an increase in synaptic connections induced by the *Nrxn2* deletion was established using dSTORM superresolution microscopy (Fig. 4). Strikingly, the added “new” synapses that were induced by the deletion of *Nrxn2* were more powerful and had a higher release probability than *Nrxn2*-containing synapses. The increase in release probability was again documented by three approaches: a decreased paired-pulse ratio (PPR) (Fig. 3E), a decreased coefficient of variation of synaptic responses (Fig. 3, F and J), and an enhanced inactivation rate of synaptic responses in the presence of MK-801 (Fig. 3, K to M). These findings suggest three conclusions.

First, *Nrxn2* performs a restrictive, and not an inductive, function in synapse assembly at least in hippocampal CA! Schaffer-collateral synapses that are a model for much of neuronal cell biology. Such a restrictive function is unprecedented—no other synaptic adhesion protein or factor, and certainly no other neurexin, is known to suppress excitatory synapse formation. The marked increase in synapse numbers induced by the *Nrxn2* deletion is unexpected given previous results, as documented in nearly a thousand papers on neurexins. Thus, our results reveal an unanticipated facet of neurexin function: to limit the number of synaptic connections.

Second, *Nrxn2* restricts synapse formation throughout life, not just during development, as demonstrated by the large increase in Schaffer collateral synaptic transmission induced by *Nrxn2* deletions in adult mice that is indistinguishable from the increase induced by the developmental *Nrxn2* deletions (Fig. 5). This result is consistent with the finding that hippocampal synapses are highly dynamic and turn over rapidly in adult mice (*51*, *52*). Thus, the hippocampus contains a large reservoir of potential synapses “in waiting” whose formation can be conditionally activated by the down-regulation of *Nrxn2*. This finding suggests a previously unknown *Nrxn2*-dependent mechanism of circuit refinement that is mediated by suppression of synapse formation and synaptic transmission controlled by *Nrxn2*.

Third, *Nrxn2* has a fundamentally different function than *Nrxn1* or *Nrxn3* despite their high degree of homology. Extensive analyses of *Nrxn1* and *Nrxn3* demonstrate that their individual deletions cause impairments in synaptic transmission in mouse and human neurons (*24*, *25*, *39*, *40*). It is unexpected that *Nrxn2* deletions do not have any of these effects, despite the fact that *Nrxn2* is highly similar to *Nrxn1* and *Nrxn3*, has the same sites of alternative splicing (except for SS#6), and is coexpressed with *Nrxn1* and *Nrxn3* in most cells. Moreover, we show that the function of *Nrxn2* is important for an animal’s behavior because the *Nrxn2* deletion phenotype in the hippocampus produces a selective but profound learning phenotype (Fig. 6).

Given that the results we present here for *Nrxn2* are quite different from what was expected, is it possible that our findings are due to systematic or technical artifacts? Because of this concern, in the present study we have examined the *Nrxn2* deletion phenotype from as many angles as possible. In view of the conceptual paradigm change prompted by our data, it seemed advisable to have strong evidence in support of the central finding that *Nrxn2* normally restricts synapse assembly. Different experimentalists studied in vivo deletions of *Nrxn2* in constitutive KO mice (Fig. 1 and figs. S1 and S2), in neuron-specific KO mice (Figs. 2 to 4 and figs. S4 and S5) and in mice in which *Nrxn2* was conditionally ablated postdevelopmentally (Fig. 5 and fig. S7). Most of these results duplicate each other, providing independent verifications. Moreover, a postsynaptic deletion of *Nrxn2* had no effect on synaptic connectivity (fig. S6).

Our results suggest that neurexins act as a "yin and yang" of synapse organization, whereby *Nrxn1* and *Nrxn3* promote the organization of various facets of synapses, whereas *Nrxn2* restricts the organization of synapses. This concept changes how we think about neurexins as central control switches of synapses, suggesting that *Nrxn2* evolved as a mechanism to limit exuberant synapse formation in neural circuits. Although the concept of a repressive function of *Nrxn2* in synapse assembly contravenes current textbook ideas, this concept is at odds only with a single previous paper in which a decrease in mEPSC (miniature excitatory postsynaptic current) frequency was detected in a line *Nrxn2* mutant mice (*44*). However, the same paper also reports a decrease in PPR consistent with an increase in release probability, and the mouse line used in this paper was generated originally in our laboratory but found to not represent an *Nrxn2* deletion. Unfortunately, the *Nrxn2* gene is difficult to target, as evidenced by the fact that our generation of *Nrxn2* cKO alleles required two selectable markers (Fig. 1A).

Our experiments have multiple limitations and raise previously unknown questions, with the major limitation being that, at present, we do not have insight into the mechanisms by which *Nrxn2* suppresses synapse assembly. How is it possible that different neurexins, which exhibit a high degree of sequence homology and similar binding activities, perform distinct functions? What signals suppress *Nrxn2* function and activate *Nrxn2*-restricted synapses physiologically, and does this activation represent a physiological mechanism of circuit plasticity? What are the molecular pathways that mediate synapse formation and/or restriction, given that all neurexins appear to bind to the same ligands? Moreover, *Nrxn2* is also highly expressed in nonneuronal cells in the brain, similar to *Nrxn1* (fig. S4) (*40*), but its expression in nonneuronal cells does not appear to drive the function that is manifested by the *Nrxn2* deletion because the *Nrxn2* deletion only in neurons had the same phenotype as the *Nrxn2* deletion in all cells (Figs. 2 to 4). As the self-avoidance of neurexin-ligand interactions demonstrated between vicinal reciprocal synapses (*53*), it is possible that neuronal *Nrxn2* interacts with glial ligands, modulating transsynaptic *Nrxn2*-ligand complexes, to regulate synapse formation and strength. Last, our findings raise evolutionary questions because invertebrates only have a single-neurexin ortholog. Do the invertebrate neurexins resemble *Nrxn1* and *Nrxn3*, and is *Nrxn2* a later evolutionary addition, as suggested by its slightly lower sequence similarity (*28*)? Addressing these questions will be a major goal in understanding how circuits are constructed for years to come.

## MATERIALS AND METHODS

### Generation of *Nrxn2* cKO mice, mouse husbandry, and genotyping

To study the role of *Nrxn2*, cKO mice were generated using the standard approaches under contract by Taconic Inc. (Hudson, NY, USA; Fig. 1) (*35*). The floxed *Nrxn2* cKO mice (*Nrxn2*^*f*/f^) contain loxP sites flanking exon 18, the first common exon of *Nrxn2*α and *Nrxn2*β whose deletion abolishes the reading frame of all *Nrxn2* transcripts [see Fig. 1B and (*16*, *35*) for details]. For all mice, we validated that Cre excised the corresponding exon(s) and blocked expression of the targeted gene using genomic and reverse transcription polymerase chain reaction (RT-PCR).

Cytomegalovirus (CMV)–Cre transgenic mice (Jackson Laboratory (JAX) stock number 006054) in which Cre is expressed throughout the whole organism (*54*) were used to generate constitutive *Nrxn2* KO mice by crossing the CMV-Cre driver mice with *Nrxn2*^*f*/f^ mice. To generate *Nrxn2* nKO mice, Baf53b-Cre transgenic mice (JAX stock number 027826) in which Cre is specifically expressed in neurons but not glia^47^ were crossing with *Nrxn2*^*f*/f^ mice. Breeding cages were maintained with crossings between CMV-Cre/+;*Nrxn2^f/f^* mice (see Fig. 1, A and B, for details) and Baf53b-Cre/+;*Nrxn2^f/f^* mice. *Nrxn2* cKO mice have been deposited to the Jackson Laboratory for distribution (JAX stock number 026683). For genotyping, mouse tails (2 mm in length) were collected in 50 mM NaOH and heated at 96°C for 10 min, then neutralized with 1 M tris-HCl (pH 7.5), and vortexed for 30 s. Samples were centrifuged at 13,000 rpm for 10 min. Supernatants were transferred to new tubes and stored at 4°C until genotyping by PCR. The following primer sequences were used for genotyping: (#1) *Nrxn2*αβ flox, 5′-CAGGGTAGGGTGTGGAATGAGGTC-3′ (forward) and 5′-GTTGAGCCTCACATCCCATTTGTCT-3′ (reverse); (#2) *Nrxn2*αβ KO, 5′-GCCTGAGGGTGGAAGCAAGGAT-3′ (forward) and 5′-CAGGGTAGGGTGTGGAATGAGGTC-3′ (reverse); (#3) *Nrxn2*αβ KO, 5′-GCCTGAGGGTGGAAGCAAGGAT-3′ (forward) and 5′-GCTCCACCTCTCCCAAGTGCTTCT-3′ (reverse); (#4) *Cre*, 5′-GCCTGCATTACCGGTCGATGCAACGA-3′ (forward) and 5′-GTGGCAGATGGCGCGGCAACACCATT-3′ (reverse).

Primer pair #1 (amplifies DNA spanning second loxP site downstream of exon 18) was used to genotype of *Nrxn2* floxed cKO mice [WT = 180 base pairs (bp); floxed cKO = 328-bp product]. Primer pair #2 amplifies DNA from 5′ of first loxP site to downstream of second loxP site and is used to detect Cre-recombined *Nrxn*2 cKO allele (product = 250 bp), and primer pair #3 amplifies DNA from 5′ of first loxP site to an exon 18 sequence and is used to detect the WT *Nrxn2* cKO allele similar to primer pair #2 (product 250 bp; can also be used for genotyping floxed allele). Primer pair #4 was used to detect Cre (product = 150 bp). Each individual tail collected from the *Nrxn2*αβ KO mice was confirmed with all primers in separate PCR reactions.

All *Nrxn2* mutant mice were maintained on a C57BL/6 background; other mice were kept on a hybrid background. All experiments were performed on littermates or equivalents; in all experiments, the experimenter was blinded to the genotype of the mice/samples being examined. All experimental procedures conformed to National Institutes of Health’s *Guidelines for the Care and Use of Laboratory Animals* and were approved by the Stanford University Administrative Panel on Laboratory Animal Care.

### Quantitative RT-PCR

Quantitative RT-PCR was performed essentially as described (*39*) using RNA isolated from hippocampus (constitutive *Nrxn2* KOs and *Nrxn2* nKOs). RNA was isolated using TRIzol reagent according to the manufacturer’s protocol (Life Technologies, Carlsbad, CA, USA). Isolated RNAs (1 ng) were subjected to target-specific RT and 16 cycles of PCR preamplification with forward and reverse primers of the detection assays. Preamplified cDNAs were then processed for real-time PCR analysis on the Biomark 96:96 Dynamic Array according to the manufacturer’s protocol (Fluidigm, South San Francisco, CA, USA). Fluorescein (FAM) dye–coupled detection assays were purchased from Integrated DNA Technologies (Coralville, IO, USA): All probes were used as previously described (*25*); please see table S1 for detailed information. To ensure the specificity of the amplification, all assays were tested with dilutions of mouse hippocampal cDNA to verify high efficiency (90 to 110%) and linear amplification [coefficient of determination (*R*^2^) > 0.96]. Transcript levels were normalized to the internal control β-actin.

### Immunohistochemistry

Mouse brain sections were prepared for immunofluorescence essentially as described (*35*, *39*). Briefly, for generation of brain sections, mice were anesthetized with isoflurane and briefly perfused with phosphate-buffered saline (PBS) and, subsequently, with 4% paraformaldehyde (PFA) in 0.1 M PBS via a perfusion pump (2 ml/min). Forebrains were dissected out and postfixed in 4% PFA for 2 hours at room temperature. The brains were then cryoprotected in 30% sucrose (in 1× PBS) for 24 hours at 4°C. Coronal brain sections (30 μm) were collected at −20°C with a cryostat (Leica, CM1050). Sections were washed with PBS and incubated in blocking solution (0.1% Triton X-100 and 5% goat serum in PBS) for 1 hour at room temperature under gentle agitation and incubated for 12 hours at 4°C with primary antibodies diluted in PBS [anti-vGluT1, 1:1000, guinea pig (Millipore); anti-vGAT, 1:500, rabbit (Millipore); anti-MAP2, 1:1000, mouse (Sigma-Aldrich) or chicken (EnCor); anti–postsynaptic density protein 95 (PSD-95), 1:1000, mouse (Thermo Fisher Scientific); and anti-Homer, 1:1000, rabbit (Millipore)] Sections were washed in PBS, treated with species-specific secondary antibodies (1:1000; Alexa 488, 545, and 633, Invitrogen) at room temperature for 1 hour, and washed again in PBS. Sections were then mounted on superfrost slides and covered with 4′,6-diamidino-2-phenylindole (DAPI) containing mounting medium (VECTASHIELD, Vector Labs). Acquisition and quantitative analyses were carried out on an average of five to eight sections per condition per mouse brain using a Nikon confocal microscope (A1Rsi). Single plane confocal images (1024 × 1024 resolution) were acquired with a 60× oil objective [PlanApo; numerical aperture (NA), 1.4]. All acquisition parameters were kept constant among different conditions within experiments. Image backgrounds were normalized, and immunoreactive elements were analyzed with Nikon analysis software automatically without operator input (object size range, 0.0 to 4.0 μm^2^).

### Immunoblotting

Experiments were performed as described (*46*). Littermate constitutive *Nrxn2* KO or *Nrxn2* nKO and WT mice were deeply anesthetized with isoflurane, and the hippocampi and cortical tissue were dissected out. Snap-frozen hippocampal and cortical tissue was Dounce-homogenized in of ice-cold complete radioimmunoprecipitation assay lysis buffer that contained 150 mM NaCl, 5 mM EDTA, 1% Triton X-100, 0.1% SDS, and 25 mM tris-HCl (pH 7.6) in addition to 1× cOmplete ULTRA protease inhibitor cocktail (Roche, catalog no. 11873580001). Lysates were incubated on ice for 20 min, followed by clarification for 20 min at 13,000 rpm at 4°C. Cleared lysates were stored at −80°C. Protein concentrations were determined using the BCA (Bicinchoninic acid assay) assay with a bovine serum albumin standard curve (Life Technologies, catalog no. 23227). Samples were diluted in Laemmli sample buffer (final concentration, 1×) containing fresh dithiothreitol and heated to 95°C for 5 min. To limit protein aggregation caused by heating multipass transmembrane proteins (i.e., vGluT1), some samples were not heated. Proteins were separated by SDS–polyacrylamide gel electrophoresis using 4 to 20% MIDI Criterion TGX precast gels (Bio-Rad). In general, proteins were transferred onto 0.2-μm pore nitrocellulose membranes for 10 min at 2.5 V using the Trans-Blot turbo transfer system (Bio-Rad). To sensitively detect neurexin levels, proteins were transferred onto nitrocellulose transfer membrane using a Criterion Blotter (Bio-Rad) with plate electrodes in ice-cold transfer buffer (25.1 mM tris, 192 mM glycine, and 20% methanol) at 80-V constant voltage for 1 hour. Membranes were blocked in 5% nonfat milk (Carnation) diluted in PBS or tris-buffered saline (TBS) for 1 hour at room temperature. Membranes were then incubated with primary antibodies diluted in TBST (TBS containing 0.1% Tween 20) overnight at 4°C. β-Actin was used as a loading control for protein quantifications. Membranes were washed three times, followed by incubation with secondary antibodies. Combinations of the following IRDye secondary antibodies were used (1:10,000 in TBST with 5% milk): IRDye 800CW donkey anti-mouse (926-32212), IRDye 680LT donkey anti-mouse (926-68022), IRDye 800CW donkey anti-rabbit (926-32213), IRDye 680LT donkey anti-rabbit (926-68023), and IRDye 680LT donkey anti–guinea pig (926-68030) from LI-COR. The Odyssey CLx imaging systems (LI-COR) was used to detect signal and was set to automatic mode for detection within linear dynamic range. Pseudo-colors were applied to the signals, and quantification was performed using Image Studio 5.2. Normalization was performed as described in the figure legends. Antibodies used were as follows: anti–pan-Nrxn (1:500; rabbit, G392, homemade), anti–pan-Nrxn (1:500; rabbit, G393, homemade), anti–pan-Nrxn (1:500; rabbit, G394, homemade), anti–pan-Nrxn (1:500; rabbit, Nrxn-Rb-Af870, Frontier Institute), anti-Nrxn1 (1:500; rabbit, 175103, Synaptic Systems), anti–pan-Nrxn (1:500; rabbit, A473, homemade), anti–pan-Nrxn (1:500; rabbit, D580, homemade); anti–neuroligin-1 (1:1000; mouse, 129111, Synaptic Systems), anti–neuroligin-2 (1:1500; mouse, 129511, Synaptic Systems), anti–neuroligin-3 (1:1000; rabbit, 129103, Synaptic Systems), anti-CASK (1:1000; mouse, 75-000, Neuromab), anti-Syt1 (synaptotagmin-1) (1:500; rabbit, W855, homemade), anti-Syt2 (1:1000; rabbit, A320, homemade), anti-synapsin (1:1000; rabbit, E028, homemade), anti-SNAP25 (synaptosomal-associated protein, 25kDa) (1:500; rabbit, 439B, homemade), anti-gephyrin (1:1000; mouse, 147111, Synaptic Systems), anti-Gad65 [1:500; mouse, mGad6-a, DHSB (Developmental Studies Hybridoma Bank)], anti–PSD-95 (1:1000; mouse, 75-028, Neuromab), anti–pan-SHANK (SH3 and multiple ankyrin repeat domains) (1:500; mouse, 75-089, Neuromab), anti-GluN1 (1:500; mouse, 54.1), anti-GluN2A (1:500; mouse, 75-288, Neuromab), anti-GluR1 (1:1000; rabbit, Ab1504, Millipore), anti-GluR2 (1:1000; mouse, 182103, Synaptic Systems), anti-vGluT1 (1:1000; rabbit, 135303, Synaptic Systems), anti–PICK-1 (protein interacting with C Kinase-1) (1:500; rabbit, U5831, homemade), anti-calbindin (1:1000; mouse, C9848, Sigma-Aldrich), and anti–β-actin (1:1000; mouse, A1978, Sigma-Aldrich). Membranes were washed with 0.05% TBST and incubated with a fluorescently labeled secondary antibodies. After washing, immunoblotting signals were detected with an Odyssey CLx Imager (LI-COR) and Odyssey software (LI-COR Biosciences). The total intensity values were calculated by Odyssey software, and each value was first normalized to actin and then normalized to control.

### Slice electrophysiology

Mice were analyzed at P38 to P45 for constitutive KO (CMV-Cre; *Nrxn2* KO) and *Nrxn*2 nKO (Baf53b-Cre; *Nrxn2* nKO) using the standard procedures (*38*, *39*, *55*). Transverse hippocampal slices from dorsal hippocampus were cut in a solution containing 228 mM sucrose, 26 mM NaHCO_3_, 11 mM glucose, 2.5 mM KCl, 1 mM NaH_2_PO_4_, 7 mM MgSO_4_, and 0.5 mM CaCl_2_ and recovered in artificial cerebrospinal fluid (ACSF) containing 119 mM NaCl, 26 mM NaHCO_3_, 11 mM glucose, 2.5 mM KCl, 1 mM NaH_2_PO_4_, 1.3 mM MgSO_4_, and 2.5 mM CaCl_2_. Whole-cell recordings were made at 30° to 32°C using an internal solution containing 140 mM CsMeSO_4_, 8 mM CsCl, 10 mM Hepes, 0.25 mM EGTA, 2 mM Mg adenosine 5′-triphosphate, 0.3 mM Na_3_ guanosine 5′-triphosphate, 0.1 mM spermine, 7 mM phosphocreatine (pH 7.25 to 7.3; osmolarity 294 to 298) for all figures except for Fig. 3 and figs. S4 and S6 in which CsCl was used instead of CsMeSO_3_. A bipolar electrode (FHC, USA) was placed in the *Stratum radiatum* or *Stratum lacunosum-moleculare* to evoke EPSCs and IPSC in CA1 pyramidal cells. Picrotoxin (50 μM) was included in extracellular ACSF in experiments examining excitatory synaptic transmission. 6-Cyano-7-nitroquinoxaline-2,3-dione (CNQX) (20 μM) or DD-AP-5 (2R)-amino-5-phosphonopentanoate (50 μM) was included in the bath to isolate pure NMDAR-EPSCs or AMPAR-EPSCs. IPSCs were pharmacologically isolated by adding blockers against AMPAR (CNQX) and NMDAR [DD-AP-5 (2R)-amino-5-phosphonopentanoate)] to the extracellular solution. Stimulation pulses were delivered every 10 s. AMPAR-EPSCs and IPSC were recorded with a holding potential of −70 mV, and NMDAR-EPSCs were recorded at +40 mV. Two pulses at different intervals (20, 50,100, 200, and 500 ms) were delivered to calculate PPRs. All drugs were obtained from Tocris (Minneapolis, MN, USA). To examine release probability, NMDAR-EPSCs were recorded for a 10-min stable baseline, and stimulation was paused for 10 min to equilibrate, while 20 μM (+)-MK-801 was added to the bath. One hundred stimuli at 0.1 Hz produced a decay curve of NMDA-EPSC amplitudes with (+)-MK-801 perfusion. The genetic manipulation of the mice is unknown to the experimenter before analyzing the data.

### dSTORM imaging

Following the procedures previously described in (*56*), dSTORM images were recorded with a Vutara SR 352 (Bruker Nanosurfaces Inc., Madison, WI) commercial microscope based on single-molecule localization biplane technology (*37*, *57*, *58*). Twenty-five–micrometer–thick hippocampal slices containing the CA1 region were prepared as described and labeled with Homer1 (1:1000; rabbit, Millipore, catalog no. ABN37) and Bassoon (1:1000; mouse, monoclonal, NeuroMab, catalog no. 75-491) primary antibodies and secondary antibodies conjugated to CF568 (1:3000; Biotium) or Alexa 647 (1:3000; Thermo Fisher Scientific). The hippocampal slices of *Nrxn2* nKO and WT mice were mounted on a coverslip coated with poly-l-lysine and placed in dSTORM buffer containing 50 mM tris-HCl (pH 8.0), 10 mM NaCl, 20 mM mercapto-ethanol, 1% β-mercaptoethanol, 10% glucose, 150 arbitrary units (AU) of glucose oxidase type VII (Sigma-Aldrich, catalog no. G2133), and 1500 AU of catalase (Sigma-Aldrich, catalog no. C40). Labeled proteins were imaged with 561- and 647-nm excitation power of 40 kW/cm^2^. Images were recorded using a 60×/1.2 NA Olympus water immersion objective and Hamamatsu Flash4 sCMOS camera with a gain set at 50 and a frame rate at 50 Hz. Data were analyzed by Vutara SRX software (version 6.04). Single molecules were identified in each frame by their brightness after removing the background. Identified molecules were localized in three dimensions by fitting the raw data in a 12-pixel by 12-pixel region of interest centered around each particle in each plane with a three-dimensional model function that was obtained from recorded datasets of fluorescent beads. Fit results were filtered by a density-based denoising algorithm to remove isolated particles and rendered as 25-nm points. The experimentally achieved image resolution of 40 nm laterally (*x*, *y*) and 70 nm axially (*z*) was determined by Fourier ring correlation.

### Stereotactic injections of viruses

*Nrxn*2 cKO mice were stereotaxically injected with viruses bilaterally into the hippocampus at P24 or P45 using a stereotaxic injection setup (David Kopf) under a general ketamine-medetomidine–induced anesthesia (*39*, *59*). A small volume (∼0.25 μl per injection site) of concentrated virus solution (10^7–8^ transduction units per ml) was injected at a slow rate (100 nl/min) using a syringe pump (Harvard Apparatus). The injection needle was withdrawn 5 min after the end of the infusion. Mice were injected at three sites per hemisphere (total = six injection sites; coordinates = (i) *X*: −1.8 mm, *Y*: 2.1 mm, *Z*: −2.0 to −1.8 mm; (ii) *X*: −2.0 mm, *Y*: 2.1 mm, *Z*: −2.0 to −1.8 mm; (iii) *X*: −2.2 mm, *Y*: 2.1 mm, *Z*: −2.0 to −1.8 mm; (iv) *X*: +1.8 mm, *Y*: 2.1 mm, *Z*: −2.0 to −1.8 mm; (v) *X*: +2.0 mm, *Y*: 2.1 mm, *Z*: −2.0 to −1.8 mm; (vi) *X*: +2.2 mm, *Y*: 2.1 mm, *Z*: −2.0 to −1.8 mm, distance measured from Bregma). Injected AAVs encoded EGFP-tagged Cre or ΔCre (for electrophysiology and behavior) or Cre–internal ribosomal entry site (IRES)– or ΔCre-IRES-tdTomato-Synaptobrevin-2 (Synapto-Tag, for morphological studies; see fig. S4A), all under control of the synapsin promoter.

### Viral production

AAVs were packaged for high-efficiency in vivo neuronal infections as described (*55*). Briefly, AAV vectors expressing the target constructs were cotransfected with AAV helper plasmid and AAV rep-cap helper plasmid into human embryonic kidney (HEK) 293 cells. At 72 hours after transfection, cells were collected and lysed by a freeze-thaw procedure and loaded onto iodixanol for centrifugation at 400,000*g* for 2 hours. The fraction with 40% iodixanol of the gradient was collected, washed, and concentrated using a 100-kDa molecular weight cutoff ultrafiltration device. The infectious virus titer was measured by infecting HEK293 cells.

### Behavioral analyses

Mice with bilateral hippocampal injection with AAV-Synapsin-Cre-GFP at P24 were collected for behavioral analysis at P60 to P80. Injection sites were confirmed with brain perfusion followed with tissue sections (80 μm) with cryostat and mounted to superfrost slides and viewed under an epifluorescence microscope (Olympus V120) after all behavioral tasks were completed. Behavioral analyses were performed on littermates as described (*57*, *60*) with the genetic manipulation of the mice unknown to the experimenter.

### Water T-maze

Spatial learning and behavioral flexibility were measured using the water T-maze (*61*). On the first day of the experiment (pretraining), mice were placed in the starting arm of the water T-maze and allowed to swim freely for 60 s. To avoid potential individual bias, the first arm that each mouse entered was recorded, and the platform was placed on the opposite arm during the learning trials. Learning training began 24 hours after pretraining, and mice were given 10 trials each day, with 10 min of rest between each trial. The time to find the platform and the number of incorrect arm entries (errors) were measured for each trial; the average time to find the platform, total number of errors, and success rate (percent of trials without an error) for each day were used for analysis. Learning training was ended and reversal training began once a mouse achieved a success rate of 80% or better for two consecutive days. For reversal training, the platform was moved to the opposite arm of the maze, and mice were given 10 trials/day for two consecutive days, following the same protocol described above.

### Open-field activity

Mice were placed in the center of a 28-cm by 28-cm force-plate actometer and allowed to freely explore for 15 min. Changes in the center of force (movement) of the mouse are monitored and analyzed using an in-house program as described previously (*62*). The following behaviors were measured: total distance traveled, spatial confinement, number of low-mobility bouts (bouts during which the center of force remained within a 30-mm-diameter circle for 10+ s), stereotypy (defined as the intensity of behavior occurring in “one place”; the movement of the center of force during LMBs (low mobility bouts) divided by the total number of LMBs), total rotations around the center of the field, and the amount of time spent in the center of the field.

### Fear conditioning

Mice were trained and tested in a conditioning chamber (18.5 cm by 18 cm by 21.5 cm) outfitted with a metal grid floor and housed inside a sound-attenuating enclosure (*61*). Training consisted of an initial 120-s exploration period to assess baseline freezing, followed by six tone-footshock pairings separated by 60-s intervals. During each paring, a 70-dB 2-kHz tone was played for 30 s and was accompanied by a 0.8-mA footshock administered during the last 2 s of the tone. The mice remained in the chamber for 30 s following the last tone-footshock pairing before being returned to their home cage. Twenty-four hours after training, the mice were returned to the conditioning chamber for 120 s to assess contextual recall. Forty-eight hours after training, the mice were placed in a new conditioning chamber modified by covering the metal grid floor with a plastic sheet, changing the color of the walls and decorating them with shapes, and by cleaning the chamber with a 1% vanilla solution. An initial 120-s exploration period was used to measure freezing in an altered context, followed by a 60-s presentation of a 70-dB 2-kHz tone to assess cued recall. Freezing behavior defined as a bout of motionless lasting 1 s or longer was measured automatically using FreezeFrame software (Coulbourn Instruments).

### Passive avoidance learning

Passive avoidance learning was performed using MED-PC IV software (Med Associates) in a shuttle box equipped with a metal grid floor and a wall separating the box into equal halves—one-half was kept in the dark and the other half was exposed to a bright light. The mice were placed in the lighted chamber, and the latency to enter the dark chamber was recorded. Once the mouse entered the dark chamber, a door separating the two chambers was closed to prevent the mouse from escaping. After a 2-s delay, a 0.3-mA footshock (3 s in duration) was administered, and the mouse was then returned to its home cage. The mice were then given a single recall trial each day 24, 48, and 72 hours after training. In the recall trials, the mice were placed in the lighted chamber for a maximum of 150 s, and the latency to enter the dark chamber was recorded.

### Accelerating rotarod

Motor learning was measured using a five-station rotarod treadmill (Med Associates) that accelerated at a rate of 4 to 40 rpm over 300 s. The mice were given 3 trials/day for 3 days, with 1-hour rest between each trial. Each trial was terminated when the mouse either fell off, completed two consecutive rotations while holding on, or after 300 s when the rotarod reached maximum speed.

### Statistical analyses

Intergroup comparisons were done by unpaired Mann-Whitney test, and each dataset was tested by Anderson-Darling test for normality. For multiple comparisons, data were analyzed with one-way or two-way analysis of variance (ANOVA) with Bonferroni’s posttest; for cumulative distributions, Kolmogorov-Smirnov tests were used. The levels of significance were set as *P < 0.05, **P < 0.01, and ***P < 0.001. Data are represented as means ± SEM.

## Supplementary Materials

### This PDF file includes:

Figs. S1 to S7 Table S1 References

## Notes

View/request a protocol for this paper from *Bio-protocol*.
