# Supplementary material for: Neurexin-2: An inhibitory neurexin that restricts excitatory synapse formation in the hippocampus
Source: Sci Adv. 2023 Jan 6;9(1):eadd8856. doi: 10.1126/sciadv.add8856 (PMC9821874; doi:10.1126/sciadv.add8856)
Supplement: Supplementary file 1 — Figs. S1 to S7 Table S1 References [file sciadv.add8856_sm.pdf]

Science Advances
AAAS

Supplementary Materials for

Neurexin-2: An inhibitory neurexin that restricts excitatory synapse formation in the hippocampus

Pei-Yi Lin *et al.*

Corresponding author: Pei-Yi Lin, pylin13@stanford.edu; Thomas C. Südhof, tcs1@stanford.edu

Sci. Adv. **9**, eadd8856 (2023)
DOI: 10.1126/sciadv.add8856

This PDF file includes:

Figs. S1 to S7
Table S1
References

SUPPLEMENTARY FIGURES and LEGENDS

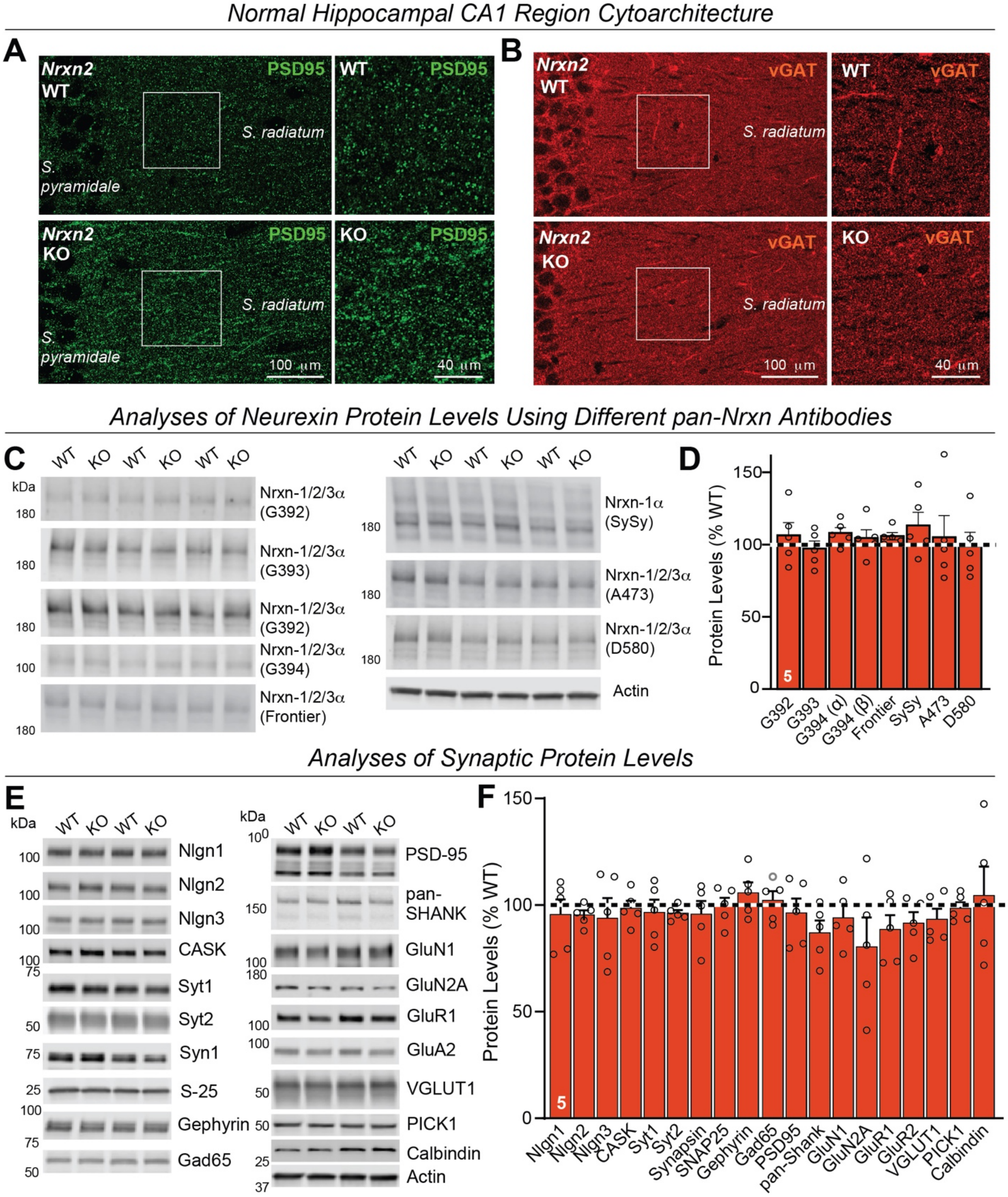

Fig. S1

The constitutive *Nrxn2* deletion does not significantly alter the general cytoarchitecture of the hippocampus or overall protein levels of neurexins and selective synaptic proteins in hippocampus (related to Fig. 1).

A & **B**, Sample cryosections of the hippocampus from constitutive *Nrxn2* KO stained for PSD95 (A) or vGAT (B) to illustrate the overall normal structure of the hippocampus in *Nrxn2* KO mice. Panels on the right exhibit the boxed section of the images on the left at a higher magnification.

C & **D**, The constitutive *Nrxn2* KO has no effect on the overall levels of neurexin proteins as analyzed by quantitative immunoblotting with 7 different primary neurexin antibodies and fluorescent secondary antibodies followed by Licor detection (**A**, representative immunoblots; **B**, summary graphs of neurexin protein levels in the hippocampus of constitutive *Nrxn2* KO mice normalized for littermate control samples). A panel of antibodies raised against conserved sequences in *Nrxn1* or *Nrxn3* C-terminal sequences were used to detect total neurexin levels. A Nrxn1-specific antibody (SySy) was used to detect Nrxn1α. Multiple neurexin antibodies were used in these experiments because neurexins have been notoriously difficult to analyze with antibodies, and the various antibodies control for each other. Moreover, no Nrxn2-specific antibody is available from any source. For quantification, protein levels are normalized to actin and expressed as a percentage of WT levels. The *Nrxn2* KO does not decrease total neurexin proteins levels presumably because all neurexin antibodies preferentially recognize Nrxn1 and react with Nrxn2 poorly as the most distantly related isoform, and because Nrxn2 expression is lower than that of Nrxn1.

E & F, Representative immunoblots (**C**) and summary graph (**D**) of synaptic protein levels in the hippocampus of littermate control and constitutive *Nrxn2* KO mice.

Numerical data are means ± SEM; 5 mice were analyzed for all quantifications. Statistical significance was assessed by Mann-Whitney tests comparing KO to control.

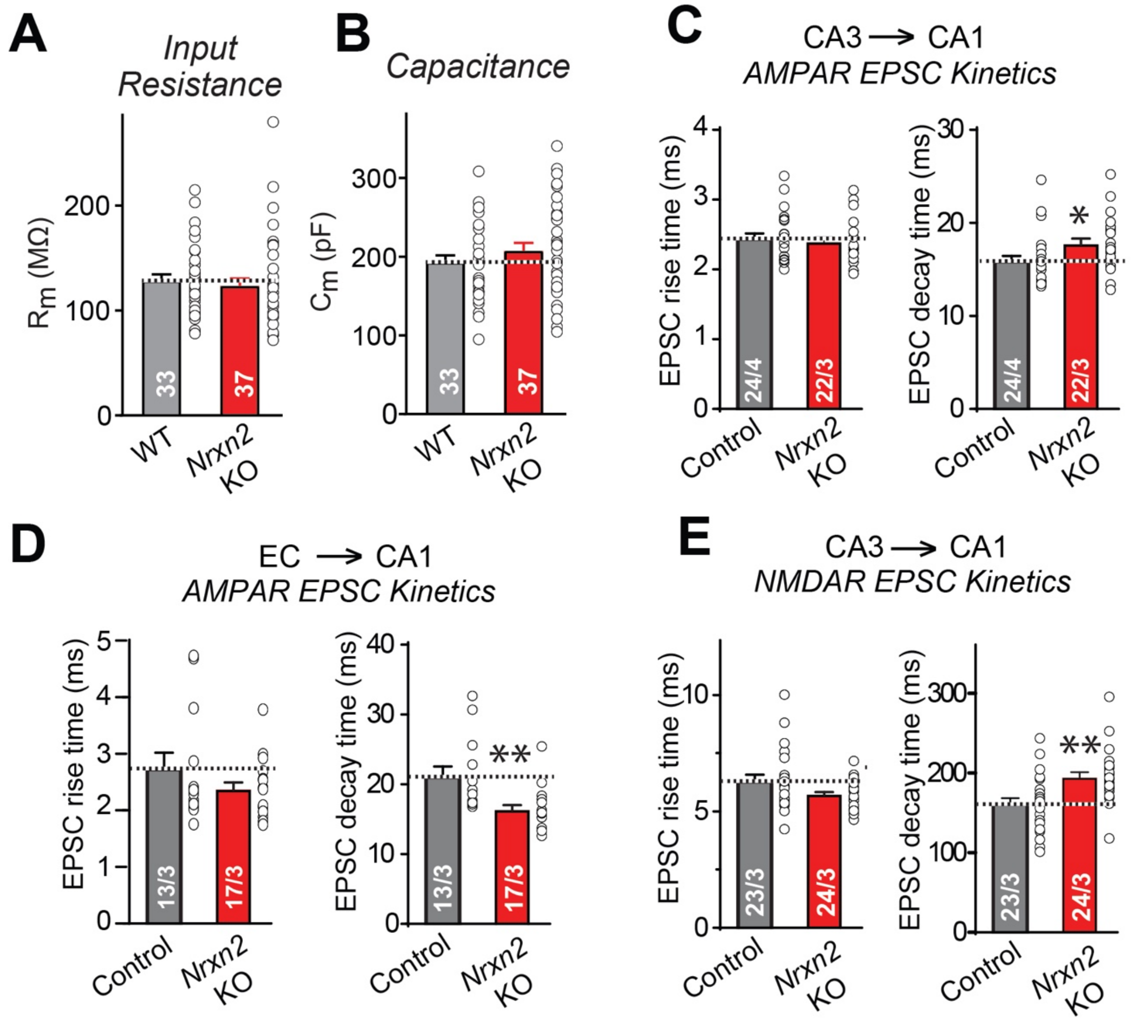

Fig. S2

The constitutive *Nrxn2* KO has no effect on the passive electrical properties of hippocampal neurons and introduces discrete modest changes the kinetics of CA3→CA1 and EC→CA1 EPSCs (related to Fig. 1).

A & B, The constitutive *Nrxn2* KO does not alter the input resistance (**A**) and capacitance (**B**) of pyramidal neurons.

C, The constitutive *Nrxn2* nKO has no effect on the rise (left panel) but decelerates the decay times (right panel) of CA3→CA1 AMPAR-EPSCs.

D, The constitutive *Nrxn2* nKO has no effect on the rise (left panel) but accelerates the decay times (right panel) of EC→CA1 AMPAR-EPSCs.

E, The constitutive *Nrxn2* nKO has no effect on the rise (left panel) but slows the decay times (right panel) of CA3→CA1 MNDAR-EPSCs.

Data shown are means ± SEMs; numbers of analyzed mice or of cells/mice are shown in the bars. Mann-Whitney tests (all bar graphs) comparing KO to WT (*P<0.05, **P<0.01, ***P<0.001).

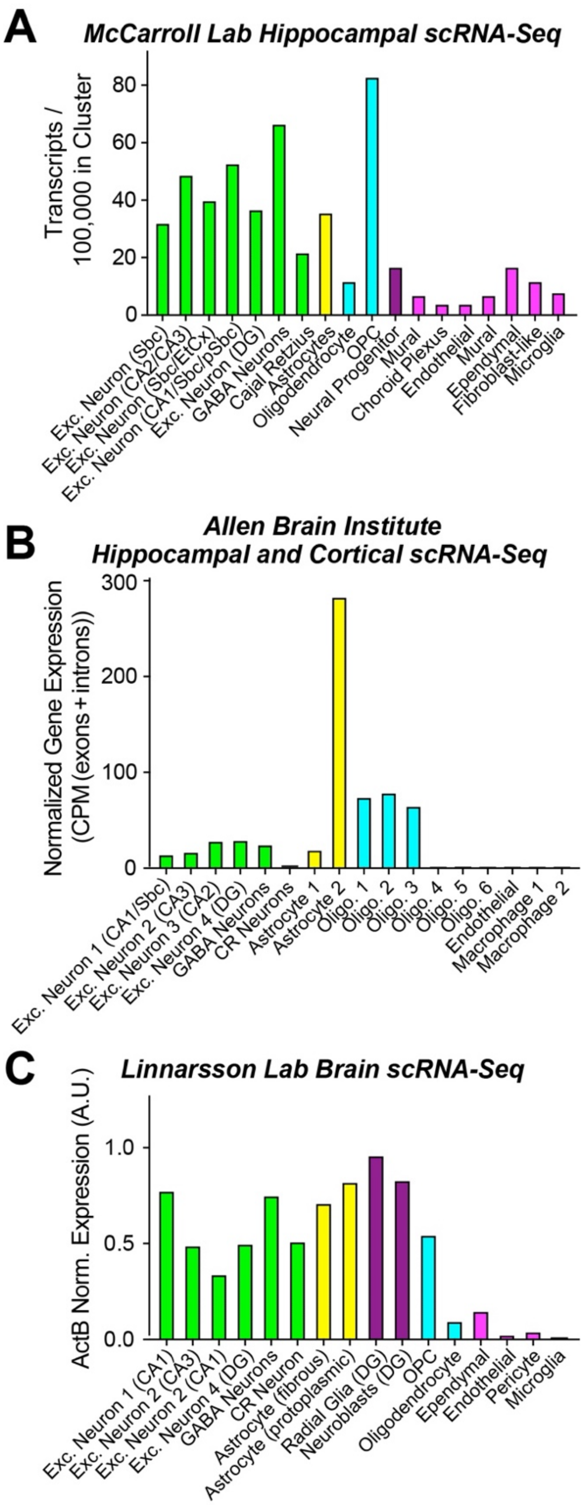

Fig. S3

Analysis of *Nrxn2* expression levels in neuronal and non-neuronal cell types of the mouse brain based on three independent single-cell RNAseq studies published by the McCarroll laboratory (63) (a), the Allen Brain Institute (64,65) (b), and the Linnarson laboratory (54) (c) reveals robust expression of *Nrxn2* in neurons and in glia in brain

A-**C**, *Nrxn2* mRNA transcript levels as analyzed by the indicated sources (see Extentded Data Refs. 1-4). Note that despite the overall similar approach (single-cell RNAseq of dissociated cells from adult mouse brain), the relative expression levels vary between studies, likely because different procedures were used for quantifying and normalizing transcripts. Despite these differences, however, all studies reveal robust *Nrxn2* mRNA levels both in astrocytes and in oligodendrocyte precursor cells (OPCs).

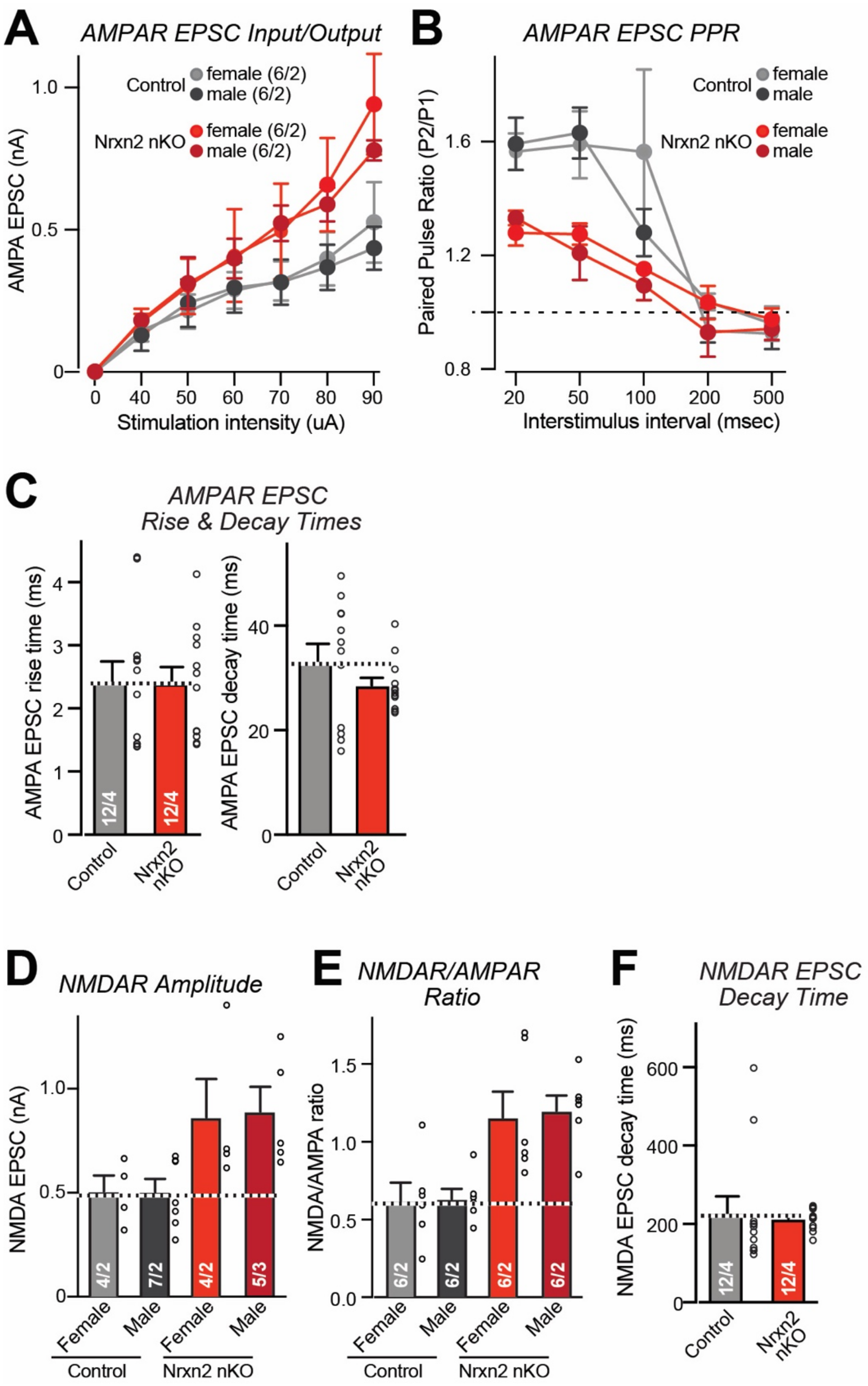

Fig. S4

The pan-neuronal deletion of *Nrxn2* (Nrxn2 nKO) causes the same synaptic phenotype as the constitutive *Nrxn2* deletion, and this synaptic phenotype is independent of the gender of the mice (related to Fig. 3).

A, Re-analyses of the data shown in Fig. **3A-3C** by examining the values separately for female and male mice reveal that the *Nrxn2* KO causes the same increase in the AMPAR-EPSC input/output curve in mice of bother genders.

B, Separate analyses of data from male and female mice for the AMPAR-EPSC paired-pulse responses in Fig. **3D-3F** reveals that female and male mice do not significantly differ in paired-pulse responses.

C, The neuron-specific *Nrxn2* nKO has no effect on the rise (left panel) and decay times (right panel) of AMPAR-EPSCs.

D & **E**, Separate analyses of data from male and female mice for the NMDAR/AMPAR ratio (**H**) and the NMDAR-EPSC amplitude (**I**) in Fig. **3G-3I** reveals that female and male mice do not significantly differ.

F, The neuron-specific *Nrxn2* nKO has no effect on the decay times of NMDAR-EPSCs.

Data shown are means ± SEMs; numbers of analyzed mice or of cells/mice are shown in the bars. Statistical assessments performed by two-way ANOVA (**A, B**) or Mann-Whitney test (all bar graphs) comparing KO to WT (*P<0.05, **P<0.01, ***P<0.001).

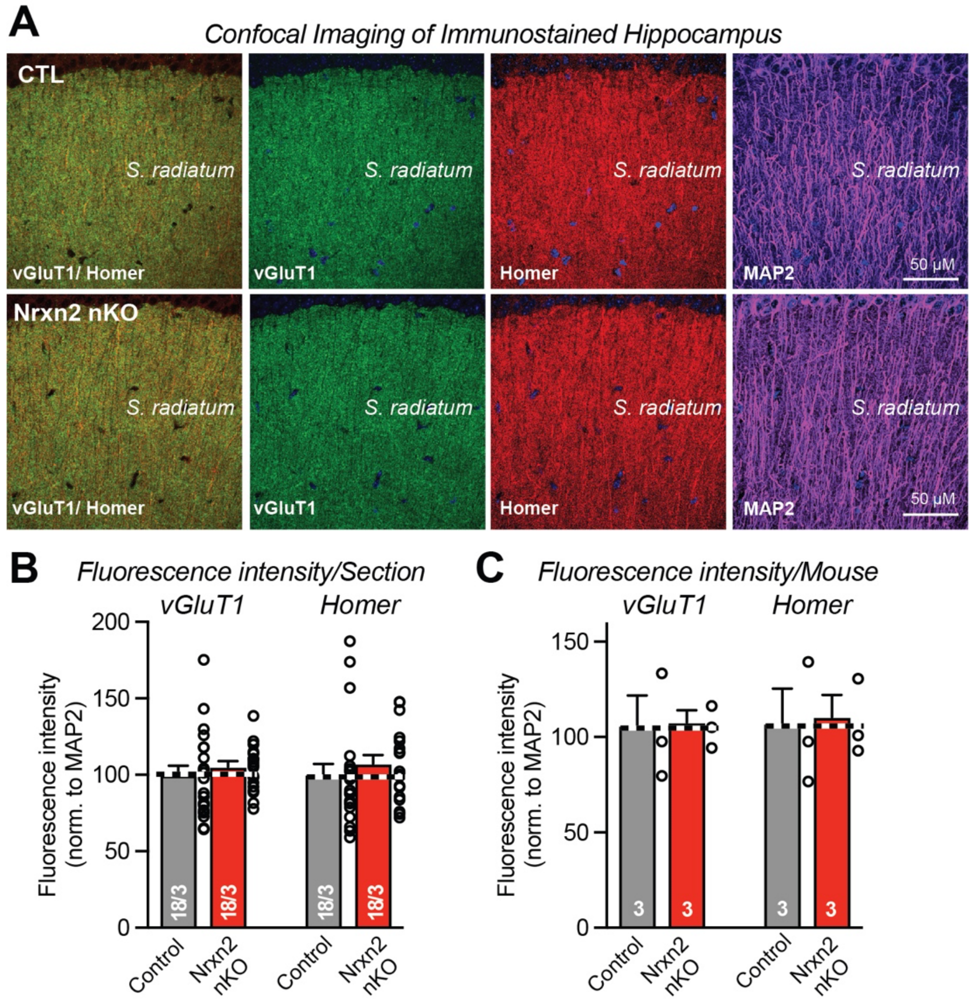

Fig. S5

Quantitative analysis of the staining intensity of the CA1 region S. radiatum from control and neuron-specific *Nrxn*2 nKO mice with antibodies to vGluT1 and Homer1, normalized to the simultaneous staining of sections with antibodies to MAP2, reveals no difference

A, Representative images of cryosections from littermate control (CTL) and *Nrxn*2 nKO mice stained for vGluT1, Homer1, and MAP2. Sections were cut from the hippocampal CA1 region, and the S. radiatum was imaged.

B & C, Summary graphs depicting the overall staining intensity for vGluT1 or Homer1 in the hippocampal sections, normalized for the MAP2 staining intensity in the same sections. Data are shown either based on the number of sections are pseudo-replicates (B) or the number of mice based on true replicates (C). Data are means ± SEMs; the number of neurons/mice analyzed are listed in the graphs. Statistical assessments were performed by Mann-Whitney test comparing the *Nrxn*2 nKO to the controls, with *p<0.05.

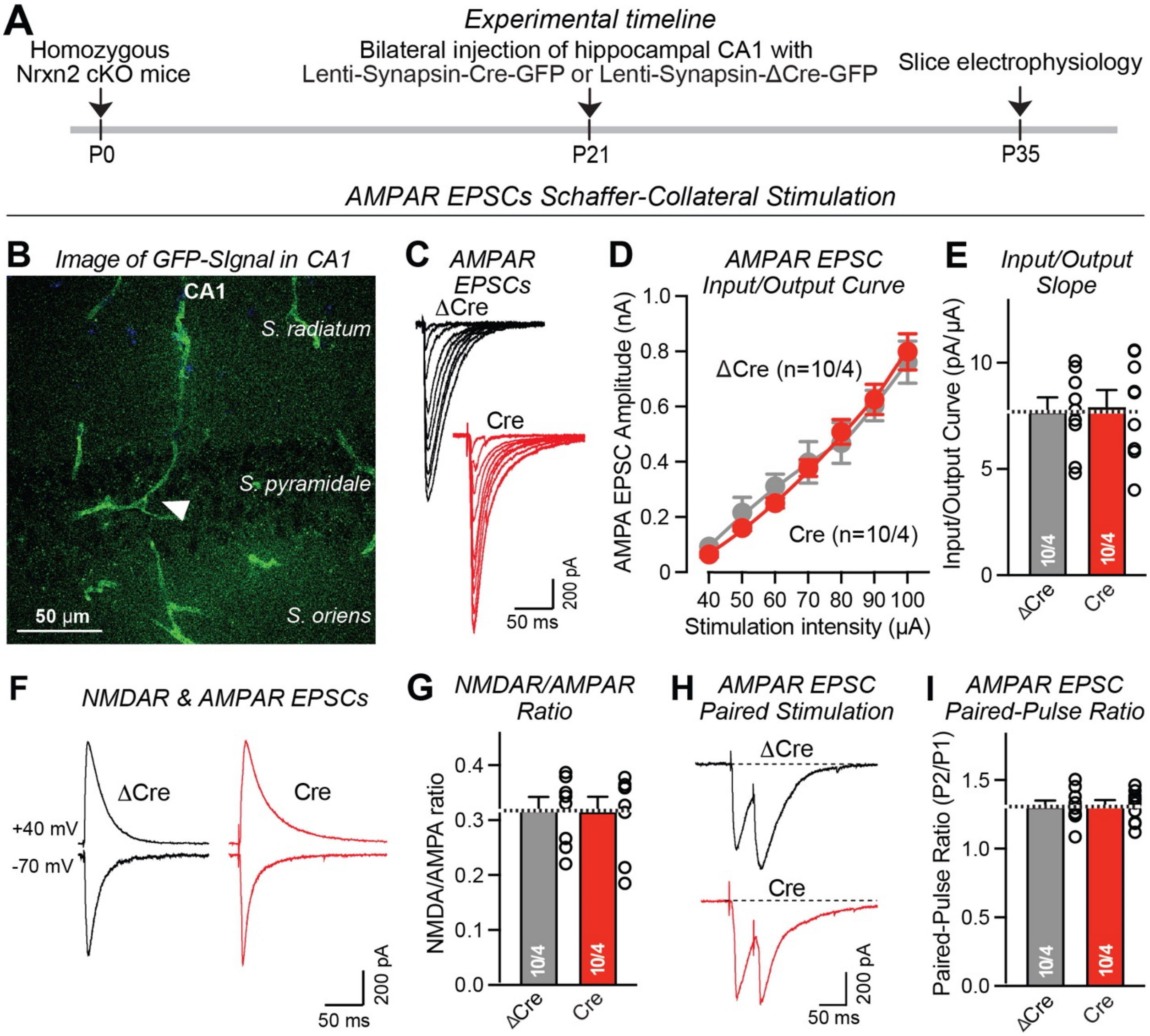

Fig. S6

Postsynaptic deletion of *Nrxn*2 fails to induce a synaptic phenotype

A, Experimental design. Sparse lentiviral infection of CA1 region neurons was used to express Cre-recombinase specifically in postsynaptic cells. Mice were analyzed two weeks later, the standard time interval employed to examine the effect of Cre-dependent deletion on synaptic properties.

B, Representative image of an acute slice used for electrophysiology experiments in which lentivirally infected cells co-express EGFP and Cre-recombinase.

C-E, Analysis of Schaffer-collateral synaptic inputs on infected control and *Nrxn*2 nKO cells fails to reveal a significant difference as analyzed in input/output measurements (C, sample traces; D, input/output curve; E, summary plot of the slope of input/output curves).

F & **G**, The *Nrxn2* nKO mutation has no effect on the NMDA/AMPA ratio (F, sample traces; G, summary graph of the NMDA/AMPA ratio)

H & **I**, The *Nrxn2* nKO mutation also has no effect on the paired-pulse ratio of AMPAR-EPSCs (F, sample traces; G, summary graph of the paired-pulse ratio)

Data shown are means ± SEMs; numbers of analyzed mice or of cells/mice are shown in the bars. Statistical assessments performed by two-way ANOVA (**A, B**) or Mann-Whitney test (all bar graphs) comparing KO to WT (*P<0.05, **P<0.01, ***P<0.001).

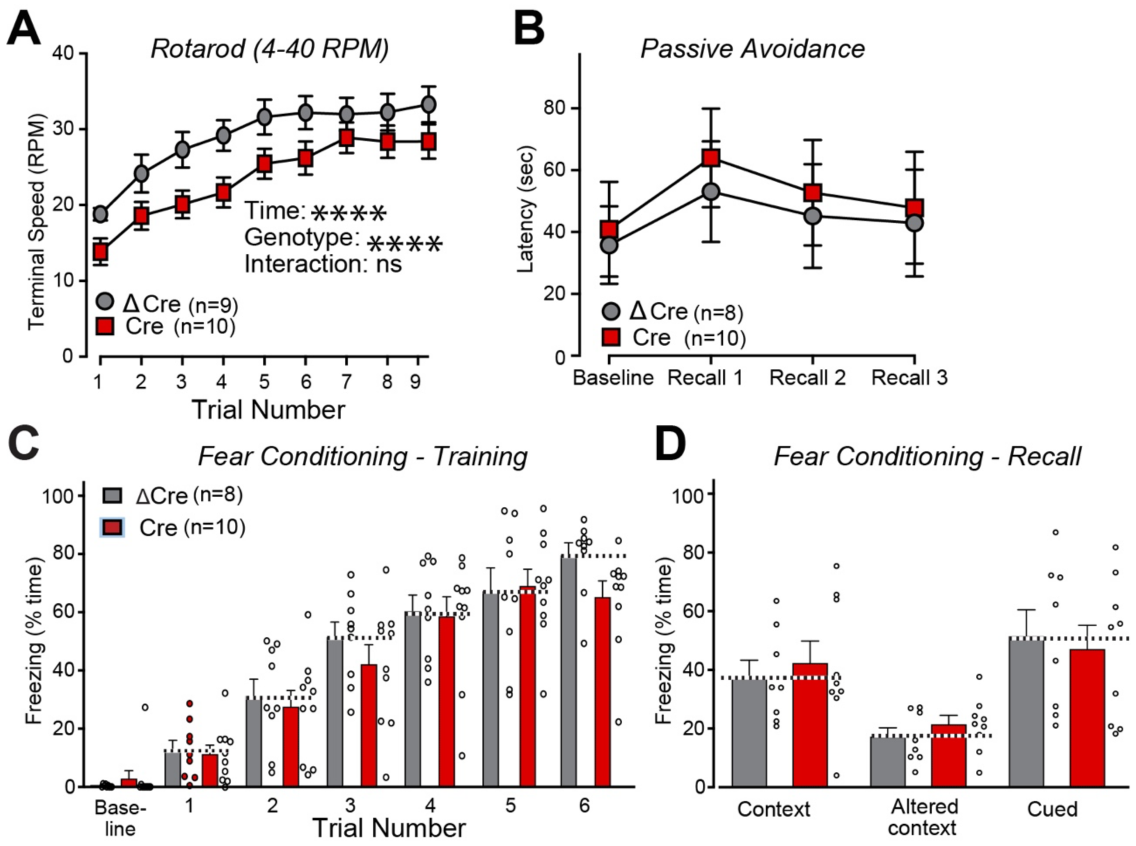

Fig. S7

Additional data on the behavioral effect of the *Nrxn2* conditional deletion in the hippocampus

A, The conditional *Nrxn2* deletion in the hippocampal formation at P24 causes a small but consistent decrease in rotarod performance, with mutant mice unable to catch up with control littermates even after extensive training.

B-D. The conditional *Nrxn2* deletion in the hippocampal formation at P24 does not alter passive avoidance (**B**) or fear conditioning (**C, D**) performance of mice.

Data shown are means ± SEMs; for all tests, 9 control (ΔCre) and 10 littermates *Nrxn2* conditionally deleted (Cre) mice were analyzed. Statistical assessments performed by Mann-Whitney test comparing KO and WT mice for all bar graphs, and by two-way ANOVA for b and c (***$p<0.001$).

Supplementary Table S1: Detailed information regarding probe usage

Fig 1B

		N number	Mean ±S.E.M	Statistics	P-value /Significance
Nrxn2	Ctrl	6	0.12±0.03	Mann-Whitney test	<0.0001/***
	Nrxn2 KO	4	0.0008±0.0005		
Nrxn2	Ctrl	6	0.10±0.04		<0.0001/***
	Nrxn2 KO	4	0.0027±0.002		
Nrxn1	Ctrl	6	0.48±0.33		0.9143/N.S.
	Nrxn2 KO	4	0.55±0.34		
Nrxn3	Ctrl	6	0.07±0.0006		0.0635/N.S.
	Nrxn2 KO	4	0.05±0.009		

Fig 1D

Stimulation intensity		N number /Mice	Mean ±S.E.M	Statistics	Interaction	Stimulation intensity factor	Group factor	P-value /Significance
30	Ctrl	27/4	26.51±10.89	Two-way ANOVA	P= 0.0015	P<0.0001	P= 0.0336	>0.9999/N.S.
	Nrxn2 KO	26/3	40.01±6.62					
40	Ctrl	27/4	63.32±20.47					0.9274/N.S.
	Nrxn2 KO	26/3	125.10±24.40					
60	Ctrl	27/4	156.79±38.12					0.1440/N.S.
	Nrxn2 KO	26/3	319.55±61.77					
80	Ctrl	27/4	252.88±55.15					0.0172/*
	Nrxn2 KO	26/3	474.65±79.95					
100	Ctrl	27/4	355.07±65.70					0.0082/**
	Nrxn2 KO	26/3	595.01±88.57					

Fig 1E

	N number /Mice	Mean ±S.E.M	Anderson-Darling test			Statistics	P-value /Significance
			Omnibus A2	P-value	Pass normality test		
Ctrl	27/4	4.91±0.87	0.2750	0.8715	Yes	Mann-Whitney test	0.0363/*
Nrxn2 KO	26/3	8.36±1.36	0.1725	0.9164	Yes		

Fig 1G

Stimulation intensity		N number /Mice	Mean ±S.E.M	Statistics	Interaction	Stimulation intensity factor	Group factor	P-value /Significance
30	Ctrl	24/3	12.33±1.33	Two-way ANOVA	P<0.0001	P<0.0001	P<0.0001	0.0417/*
	Nrxn2 KO	24/3	43.75±7.89					
40	Ctrl	24/3	27.38±2.81					<0.0001/***
	Nrxn2 KO	24/3	88.81±14.28					
60	Ctrl	24/3	73.57±8.95					<0.0001/***
	Nrxn2 KO	24/3	174.59±20.87					
80	Ctrl	24/3	106.21±11.03					<0.0001/***
	Nrxn2 KO	24/3	247.58±27.16					
100	Ctrl	24/3	139.31±13.21					<0.0001/***
	Nrxn2 KO	24/3	313.59±31.96					

Fig 1H

	N number /Mice	Mean ±S.E.M	Anderson-Darling test			Statistics	P-value /Significance
			Omnibus A2	P-value	Pass normality test		
Ctrl	24/3	1.861±0.18	0.3579	0.4081	Yes	Mann-Whitney test	<0.0001/***
Nrxn2 KO	24/3	3.90±0.37	0.3687	0.3938	Yes		

Fig 1J

Stimulation intensity		N number /Mice	Mean ±S.E.M	Statistics	Interaction	Stimulation intensity factor	Group factor	P-value /Significance
30	Ctrl	13/3	69.24±19.65	Two-way ANOVA	P= 0.7361	P<0.0001	P= 0.5353	>0.9999/N.S.
	Nrxn2 KO	20/3	74.70±20.21					
40	Ctrl	13/3	174.05±44.63					>0.9999/N.S.
	Nrxn2 KO	20/3	171.46±38.85					
60	Ctrl	13/3	430.23±97.11					0.9950/N.S.
	Nrxn2 KO	20/3	399.31±75.17					
80	Ctrl	13/3	689.92±143.40					0.8163/N.S.
	Nrxn2 KO	20/3	585.75±97.16					
100	Ctrl	13/3	859.92±143.40					0.9188/N.S.
	Nrxn2 KO	20/3	788.79±124.42					

Fig 1K

	N number /Mice	Mean ±S.E.M	Anderson-Darling test			Statistics	P-value /Significance
			Omnibus A2	P-value	Pass normality test		
Ctrl	13/3	11.74±2.14	0.4962	0.1744	Yes	Mann-Whitney test	0.5990/N.S.
Nrxn2 KO	20/3	10.37±1.55	0.5286	0.1555	Yes		

Fig 2B

	Offspring Ratio	
	Control	Nrxn2 nKO
Female	58.7	41.3
male	45.83	54.17

Fig 2C

		N number	Mean ±S.E.M	Anderson-Darling test			Statistics	P-value /Significance
				Omnibus A2	P-value	Pass normality test		
Female	Ctrl	27	13.11±0.76	0.2555	0.6963	Yes	Mann-Whitney test	0.0002/***
	Nrxn2 nKO	19	9.03±0.56	0.2665	0.6487	Yes		
Male	Ctrl	22	13.92±1.02	0.8787	0.6124	Yes	Mann-Whitney test	0.0006/***
	Nrxn2 nKO	26	9.73±20.60	0.2589	0.6872	Yes		

Fig 2D

Hippocampus	N number	Mean ±S.E.M	Statistics	P-value /Significance
Nrxn1	4	1.10±0.11		
Nrxn3	4	1.07±0.09		
Nrxn2a	4	0.41±0.07		
Nrxn2b	4	0.09±0.01		
vGluT1	4	1.00±0.03		

Fig 2D

Cortex	N number	Mean ±S.E.M	Statistics	P-value /Significance
Nrxn1	4	1.13±0.04		
Nrxn3	4	1.07±0.09		
Nrxn2a	4	0.23±0.08		
Nrxn2b	4	0.05±0.02		
vGluT1	4	1.08±0.06		

Fig 2F

Cortical	**N number /Mice**	**Mean ±S.E.M**	**Statistics**	**P-value /Significance**
Nrxn's	4	93.22±3.94		
PSD95	4	100.9±5.24		
GluA2	4	111.8±12.53		
vGluT1	4	104.2±3.73		
SNAP25	4	91.43±6.34		
Syb2	4	98.13±2.21		

Fig 3B

**Stimulation intensity**		**N number /Mice**	**Mean ±S.E.M**	**Statistics**	**Interaction**	**Stimulation intensity factor**	**Group factor**	**P-value /Significance**
40	Ctrl	12/4	125.9±24.87	Two-way ANOVA	p=0.0081	P<0.0001	P<0.0001	0.9932/N.S.
	Nrxn2 nKO	12/4	168.7±15.83					
50	Ctrl	12/4	205.9±37.26					0.7246/N.S.
	Nrxn2 nKO	12/4	296.3±42.63					
60	Ctrl	12/4	261.6±40.99					0.3219/N.S.
	Nrxn2 nKO	12/4	398.6±55.22					
70	Ctrl	12/4	288.1±40.01					0.0084/**
	Nrxn2 nKO	12/4	491.4±56.34					
80	Ctrl	12/4	348.6±48.06					0.0055/**
	Nrxn2 nKO	12/4	574.9±69.82					
90	Ctrl	12/4	395.7±64.18					0.0007/***
	Nrxn2 nKO	12/4	875.2±80.64					

Fig 3C

	N number /Mice	Mean ±S.E.M	Anderson-Darling test			Statistics	P-value /Significance
			Omnibus A2	P-value	Pass normality test		
Ctrl	12/4	4.09±0.49	0.4433	0.2364	Yes	Mann-Whitney test	0.0051/**
Nrxn2 nKO	12/4	7.05±0.79	0.5215	0.1460	Yes		

Fig 3E

Interstim. Interval		N number /Mice	Mean ±S.E.M	Statistics	Interaction	Interstim. interval factor	Group factor	P-value /Significance
20	Ctrl	12/4	1.58±0.05	Two-way ANOVA	P= 0.0045	P<0.0001	P<0.0001	0.0118/*
	Nrxn2 nKO	12/4	1.31±0.02					
50	Ctrl	12/4	1.61±0.07					0.0003/***
	Nrxn2 nKO	12/4	1.24±0.05					
100	Ctrl	12/4	1.42±0.15					0.0029/**
	Nrxn2 nKO	12/4	1.12±0.03					
200	Ctrl	12/4	0.99±0.03					>0.9999/N.S.
	Nrxn2 nKO	12/4	0.98±0.05					
500	Ctrl	12/4	0.94±0.03					>0.9999/N.S.
	Nrxn2 nKO	12/4	0.97±0.03					

Fig 3F

	N number /Mice	Mean ±S.E.M	Anderson-Darling test			Statistics	P-value /Significance
			Omnibus A2	P-value	Pass normality test		
Ctrl	12/4	10.96±2.42	0.2270	0.2399	Yes	Mann-Whitney test	0.0479/*
Nrxn2 nKO	12/4	5.45±0.74	0.4657	0.2062	Yes		

Fig 3H

	N number /Mice	Mean ±S.E.M	Anderson-Darling test			Statistics	P-value /Significance
			Omnibus A2	P-value	Pass normality test		
Ctrl	12/4	0.63±0.06	0.4859	0.1822	Yes	Mann-Whitney test	0.0018/**
Nrxn2 nKO	12/4	1.18±0.09	0.3290	0.4614	Yes		

Fig 3I

	N number /Mice	Mean ±S.E.M	Anderson-Darling test			Statistics	P-value /Significance
			Omnibus A2	P-value	Pass normality test		
Ctrl	11/4	505.6±45.74	0.4449	0.1989	Yes	Mann-Whitney test	<0.0001/***
Nrxn2 nKO	9/5	878.3±97.89	0.3927	0.3711	Yes		

Fig 3J

	N number /Mice	Mean ±S.E.M	Anderson-Darling test			Statistics	P-value /Significance
			Omnibus A2	P-value	Pass normality test		
Ctrl	12/4	10.05±1.93	0.6116	0.0852	Yes	Mann-Whitney test	0.00159/**
Nrxn2 nKO	12/4	4.37±0.76	0.4313	0.2539	Yes		

Fig 3L

Stimulus number		N number /Mice	Mean ±S.E.M	Statistics	P-value /Significance
10	Ctrl	11/4	78.58±1.82	Comparison of fits	<0.0001/***
	Nrxn2 nKO	9/5	70.12±2.01		
20	Ctrl	11/4	65.49±3.48		
	Nrxn2 nKO	9/5	51.2±2.36		
30	Ctrl	11/4	56.16±3.43		
	Nrxn2 nKO	9/5	38.1±2.78		
40	Ctrl	11/4	45.84±4.00		

	Nrxn2 nKO	9/5	33.71±2.60		
50	Ctrl	11/4	40.53±3.51		
	Nrxn2 nKO	9/5	30.12±2.80		
60	Ctrl	11/4	35.19±3.23		
	Nrxn2 nKO	9/5	27.25±2.53		
70	Ctrl	11/4	31.65±3.2		
	Nrxn2 nKO	9/5	26.1±2.49		
80	Ctrl	11/4	30.17±2.84		
	Nrxn2 nKO	9/5	25.2±2.23		
90	Ctrl	11/4	27.69±2.69		
	Nrxn2 nKO	9/5	25.16±2.32		
100	Ctrl	11/4	24.73±2.67		
	Nrxn2 nKO	9/5	23.47±1.90		

Fig 3M

	N number /Mice	Mean ±S.E.M	Anderson-Darling test			Statistics	P-value /Significance
			Omnibus A2	P-value	Pass normality test		
Ctrl	11/4	39.11±3.81	0.2492	0.8828	Yes	Mann-Whitney test	0.0003/***
Nrxn2 nKO	9/5	19.55±0.94	0.2187	0.7673	Yes		

Fig 3O

Stimulation intensity		N number /Mice	Mean ±S.E.M	Statistics	Interaction	Stimulation intensity factor	Group factor	P-value /Significance
30	Ctrl	10/3	109±44.88	Two-way ANOVA	P<0.0001	P<0.0001	P<0.0001	0.9984/N.S.
	Nrxn2 nKO	10/3	162±26.67					
40	Ctrl	10/3	276.6±60.5					0.9682/N.S.
	Nrxn2 nKO	10/3	319.8±77.53					
50	Ctrl	10/3	461.4±91.22					>0.9999/N.S.
	Nrxn2 nKO	10/3	480.1±102					

60	Ctrl	10/3	691±117.2					>0.9999/N.S.
	Nrxn2 nKO	10/3	686.8±106.3					
70	Ctrl	10/3	793±138.4					0.9994/N.S.
	Nrxn2 nKO	10/3	848.8±116.1					
80	Ctrl	10/3	953.3±156.3					0.9998/N.S.
	Nrxn2 nKO	10/3	1003±127.1					
90	Ctrl	10/3	1087±163.9					0.9989/N.S.
	Nrxn2 nKO	10/3	1147±152.2					
100	Ctrl	10/3	1247±231.9					0.9990/N.S.
	Nrxn2 nKO	10/3	1327±174.4					

Fig 3Q

	N number /Mice	Mean ±S.E.M	Anderson-Darling test			Statistics	P-value /Significance
			Omnibus A2	P-value	Pass normality test		
Ctrl	10/3	0.61±0.03	0.2052	0.8210	Yes	Mann-Whitney test	>0.9999/N.S.
Nrxn2 nKO	10/3	0.62±0.03	0.1584	0.9269	Yes		

Fig 4C

	N number /Mice	Mean ±S.E.M	Anderson-Darling test			Statistics	P-value /Significance
			Omnibus A2	P-value	Pass normality test		
Ctrl	-/6	71.76±2.85	0.8409	0.1326	Yes	Mann-Whitney test	0.8638/N.S.
Nrxn2 nKO	-/6	71.09±2.49	0.9414	0.6704	Yes		

Fig 4D

		N number /Mice	Mean ±S.E.M	Anderson-Darling test			Statistics	P-value /Significance
				Omnibus A2	P-value	Pass normality test		
Bassoon	Ctrl	33/6	0.45±0.05	0.4581	0.2478	Yes	Mann-Whitney test	<0.0001/***
	Nrxn2 nKO	37/6	0.85±0.08	0.8748	0.2843	Yes		
Homer1	Ctrl	33/6	0.56±0.07	0.5821	0.0544	Yes	Mann-Whitney test	<0.0001/***
	Nrxn2 nKO	37/6	1.01±0.03	0.7333	0.0511	Yes		

Fig 4E

		N number /Mice	Mean ±S.E.M	Anderson-Darling test			Statistics	P-value /Significance
				Omnibus A2	P-value	Pass normality test		
Bassoon	Ctrl	-/6	0.43±0.09	0.8879	0.3074	Yes	Mann-Whitney test	0.0338/*
	Nrxn2 nKO	-/6	0.83±0.14	0.8997	0.3722	Yes		
Homer1	Ctrl	-/6	0.50±0.16	0.9358	0.6260	Yes	Mann-Whitney test	0.0206/*
	Nrxn2 nKO	-/6	0.99±0.08	0.8721	0.2345	Yes		

Fig 4F

Cluster Volume		N number /Mice	Mean ±S.E.M	Anderson-Darling test			Statistics	P-value /Significance
				Omnibus A2	P-value	Pass normality test		
Bassoon	Ctrl	33/6	0.004±0.0006	0.4581	0.2478	Yes	Mann-Whitney test	0.5361/N.S.
	Nrxn2 nKO	37/6	0.004±0.0003	0.8748	0.2843	Yes		
Homer1	Ctrl	33/6	0.016±0.002	0.5821	0.0544	Yes	Mann-Whitney test	<0.0001/***
	Nrxn2 nKO	37/6	0.028±0.001	0.7333	0.0511	Yes		

Cluster Size		N number /Mice	Mean ±S.E.M	Anderson-Darling test			Statistics	P-value /Significance
				**Omnibus A2**	**P-value**	**Pass normality test**		
Bassoon	Ctrl	33/6	95.52±2.96	0.8716	0.0957	Yes	Mann-Whitney test	0.8768/N.S.
	Nrxn2 nKO	37/6	95.04±1.22	0.8610	0.0934	Yes		
Homer1	Ctrl	33/6	133.2±3.68	0.9449	0.0664	Yes	Mann-Whitney test	<0.0001/***
	Nrxn2 nKO	37/6	158±2.63	0.5636	0.1347	Yes		

Fig 4G

Particles per Cluster		N number /Mice	Mean ±S.E.M	Anderson-Darling test			Statistics	P-value /Significance
				**Omnibus A2**	**P-value**	**Pass normality test**		
Bassoon	Ctrl	33/6	66.62±6.85	0.8289	0.2552	Yes	Mann-Whitney test	0.4828/N.S.
	Nrxn2 nKO	37/6	61.23±3.36	0.1335	0.0938	Yes		
Homer1	Ctrl	33/6	123.9±14.55	0.8289	0.1323	Yes	Mann-Whitney test	<0.0001/***
	Nrxn2 nKO	37/6	224.2±14.99	0.3758	0.3949	Yes		
Particle Density		**N number /Mice**	**Mean ±S.E.M**	**Anderson-Darling test**			**Statistics**	**P-value /Significance**
				**Omnibus A2**	**P-value**	**Pass normality test**		
Bassoon	Ctrl	33/6	$5.47*10^{-5}\pm2.96*10^{-6}$	0.9083	0.1155	Yes	Mann-Whitney test	0.4828/N.S.
	Nrxn2 nKO	37/6	$5.47*10^{-5}\pm1.62*10^{-6}$	0.9221	0.1447	Yes		
Homer1	Ctrl	33/6	$9.97*10^{-5}\pm1.87*10^{-6}$	0.6155	0.1003	Yes	Mann-Whitney test	0.0023/**
	Nrxn2 nKO	37/6	$10.68*10^{-5}\pm1.20*10^{-6}$	0.9367	0.0544	Yes		

Fig 5C

Stimulation intensity		N number /Mice	Mean ±S.E.M	Statistics	Interaction	Stimulation intensity factor	Group factor	P-value /Significance
30	ΔCre	20/3	26.7±7.84		P<0.0001	P<0.0001	P=0.001	0.3746/N.S.

	Cre	20/3	76.54±19.88	Two-way ANOVA				
40	ΔCre	20/3	48.49±12.34					0.0018/**
	Cre	20/3	156.87±19.88					
60	ΔCre	20/3	132.12±26.84					<0.0001/***
	Cre	20/3	293.91±34.58					
80	ΔCre	20/3	217.38±43.96					<0.0001/***
	Cre	20/3	441.04±47.12					
100	ΔCre	20/3	299.86±54.65					<0.0001/***
	Cre	20/3	568.18±61.03					

Fig 5D

	N number /Mice	Mean ±S.E.M	Anderson-Darling test			Statistics	P-value /Significance
			Omnibus A2	P-value	Pass normality test		
ΔCre	20/3	3.94±0.69	0.7556	0.1983	Yes	Mann-Whitney test	0.0044/**
Cre	20/3	7.03±0.76	0.8645	0.1767	Yes		

Fig 5E

		N /Mice	Mean ±S.E.M	Anderson-Darling test			Statistics	P-value /Significance
				Omnibus A2	P-value	Pass normality test		
EPSC rise time	ΔCre	18/3	2.35±0.10	0.8632	0.0925	Yes	Mann-Whitney test	0.1723/N.S.
	Cre	11/3	2.58±0.12	0.9471	0.0600	Yes		
EPSC decay time	ΔCre	18/3	16.9±1.02	0.9585	0.4590	Yes	Mann-Whitney test	0.6086/N.S.
	Cre	11/3	17.63±0.61	0.3649	0.4285	Yes		

Fig 5G

Stimulation intensity		N number /Mice	Mean ±S.E.M	Statistics	Interaction	Stimulation intensity factor	Group factor	P-value /Significance
30	ΔCre	16/3	12.60±2.11	Two-way ANOVA	P<0.0001	P<0.0001	P=0.0015	0.9841/N.S.
	Cre	20/4	29.12±5.86					

40	ΔCre	16/3	35.38±7.61					0.5549/N.S.
	Cre	20/4	74.33±12.07					
60	ΔCre	16/3	102.21±21.08					0.0009/***
	Cre	20/4	201.39±23.40					
80	ΔCre	16/3	166.18±26.97					<0.0001/***
	Cre	20/4	284.11±30.69					
100	ΔCre	16/3	221.67±30.46					<0.0001/***
	Cre	20/4	348.86±35.92					

Fig 5H

	N number /Mice	Mean ±S.E.M	Anderson-Darling test			Statistics	P-value /Significance
			Omnibus A2	P-value	Pass normality test		
ΔCre	16/3	3.24±0.38	0.2407	0.7472	Yes	Mann-Whitney test	0.0355/*
Cre	20/4	4.66±0.49	0.4836	0.2205	Yes		

Fig 5I

		N /Mice	Mean ±S.E.M	Anderson-Darling test			Statistics	P-value /Significance
				Omnibus A2	P-value	Pass normality test		
EPSC rise time	ΔCre	16/3	6.77±0.14	0.9248	0.6298	Yes	Mann-Whitney test	0.1354/N.S.
	Cre	18/4	6.23±0.31	0.2373	0.7588	Yes		
EPSC decay time	ΔCre	16/3	184.6±10.08	0.1931	0.8837	Yes	Mann-Whitney test	<0.0001/***
	Cre	18/4	273.3±13.62	0.6977	0.0595	Yes		

Fig 6A-F

		N number	Mean ±S.E.M	Anderson-Darling test			Statistics	P-value /Significance
				Omnibus A2	P-value	Pass normality test		
Distance Traveled	ΔCre	9	806.91±37.96	0.8887	0.1936	Yes	Mann-Whitney test	0.8143N.S.
	Cre	10	822.99±53.94	0.4185	0.2541	Yes		

Spatial Confinement	ΔCre	9	93.05±1.72	0.6637	0.0576	Yes	Mann-Whitney test	0.7199/N.S.
	Cre	10	90.99±5.13	0.5019	0.1499	Yes		
Low-mobility Bouts	ΔCre	9	6.22±1.52	0.3253	0.4558	Yes	Mann-Whitney test	0.2765/N.S.
	Cre	10	9.03±31.92	0.9283	0.4316	Yes		
Stereotype	ΔCre	9	55.09±1.78	0.9321	0.5018	Yes	Mann-Whitney test	0.1497/N.S.
	Cre	10	65.22±6.15	0.2799	0.5533	Yes		
Rotations	ΔCre	9	10.03±2.02	0.8348	0.0505	Yes	Mann-Whitney test	0.7780/N.S.
	Cre	10	10.60±3.97	0.6398	0.0636	Yes		
Time in Center	ΔCre	9	2.47±0.27	0.4129	0.2712	Yes	Mann-Whitney test	0.4885/N.S.
	Cre	10	2.01±0.42	0.2807	0.5509	Yes		

Fig 6G

		N number	Mean ±S.E.M	Anderson-Darling test			Statistics	P-value /Significance
				**Omnibus A2**	**P-value**	**Pass normality test**		
Day 1	ΔCre	9	5.74±1.68	0.8470	0.0534	Yes	Mann-Whitney test	0.2907/N.S.
	Cre	10	8.97±2.36	0.5931	0.0894	Yes		
Day 2	ΔCre	9	2.66±0.49	0.8882	0.1616	Yes	Mann-Whitney test	0.2480/N.S.
	Cre	10	3.55±0.44	0.4572	0.2067	Yes		

Fig 6H

		N number	Mean ±S.E.M	Anderson-Darling test			Statistics	P-value /Significance
				**Omnibus A2**	**P-value**	**Pass normality test**		
Day 1	ΔCre	9	2.44±0.73	0.3342	0.4224	Yes	Mann-Whitney test	0.7220/N.S.
	Cre	10	2.80±0.67	0.8886	0.6413	Yes		
Day 2	ΔCre	9	0.44±0.44	-	-	-	Mann-Whitney test	0.9269/N.S.
	Cre	10	0.5±0.4	-	-	-		

Fig 6I

		N number	Mean ±S.E.M	Anderson-Darling test			Statistics	P-value /Significance
				Omnibus A2	P-value	Pass normality test		
Day 1	ΔCre	9	81.11±5.12	0.4812	0.1712	Yes	Mann-Whitney test	0.7873/N.S.
	Cre	10	79±5.67	0.4165	0.2653	Yes		
Day 2	ΔCre	9	97.78±2.22	-	-	-	Mann-Whitney test	0.6503/N.S.
	Cre	10	96±3.06	-	-	-		

Fig 6J

	N number /Mice	Mean ±S.E.M	Anderson-Darling test			Statistics	P-value /Significance
			Omnibus A2	P-value	Pass normality test		
ΔCre	9	2.22±0.15	-	-	-	Mann-Whitney test	0.2326/N.S.
Cre	10	2.5±0.17	-	-	-		

Fig 6K

		N number	Mean ±S.E.M	Anderson-Darling test			Statistics	P-value /Significance
				Omnibus A2	P-value	Pass normality test		
Day 1	ΔCre	9	3.86±0.77	0.9190	0.3486	Yes	Mann-Whitney test	0.0469/*
	Cre	10	5.74±0.79	0.3513	0.3918	Yes		
Day 2	ΔCre	9	2.1±0.17	0.8479	0.0549	Yes	Mann-Whitney test	0.9204/N.S.
	Cre	10	2.4±0.13	0.6496	0.0628	Yes		

Fig 6L

		N number	Mean ±S.E.M	Anderson-Darling test			Statistics	P-value /Significance
				Omnibus A2	P-value	Pass normality test		
Day 1	ΔCre	9	1.89±0.82	0.9347	0.0910	Yes	Mann-Whitney test	0.0248/*
	Cre	10	4.7±0.79	0.7699	0.0714	Yes		
Day 2	ΔCre	9	0	-	-	-	Mann-Whitney test	0.2012/N.S.
	Cre	10	0.3±0.21	-	-	-		

Fig 6M

		N number	Mean ±S.E.M	Anderson-Darling test			Statistics	P-value /Significance
				Omnibus A2	P-value	Pass normality test		
Day 1	ΔCre	9	81.11±8.07	0.8234	0.2177	Yes	Mann-Whitney test	0.0338/*
	Cre	10	56±7.33	0.8531	0.1689	Yes		
Day 2	ΔCre	9	100±0	-	-	-	Mann-Whitney test	0.9129/N.S.
	Cre	10	97±2.13	-	-	-		

Fig 6N

	N number /Mice	Mean ±S.E.M	Anderson-Darling test			Statistics	P-value /Significance
			Omnibus A2	P-value	Pass normality test		
ΔCre	9	2.89±0.80	0.8701	0.1001	Yes	Mann-Whitney test	0.005/**
Cre	10	7.7±1.21	0.5927	0.0896	Yes		

Supplementary Fig 1D

Hippocampal	N number /Mice	Mean ±S.E.M	Statistics	P-value /Significance
G392	5	106.4±8.84		
G393	5	97.05±5.48		
G394 (a)	5	108.1±3.81		
G394 (b)	5	104.5±5.89		
Frontier	5	105.9±2.55		
Sysy	5	113.1±9.17		
A473	5	105.0±15.05		
D580	5	98.72±9.86		

Supplementary Fig 1F

Cortical	N number /Mice	Mean ±S.E.M	Statistics	P-value /Significance
Nlgn1	5	95.52±7.19		
Nlgn2	5	95.15±2.29		
Nlgn3	5	93.79±9.37		
CASK	5	98.57±3.52		
Syt1	5	96.56±5.97		
Syt2	5	96.11±1.51		
Synapsin	5	95.65±6.33		
SNAP25	5	99.54±3.92		
Gephyrin	5	105.4±4.92		
Gad65	5	102.2±4.29		
PSD95	5	96.29±6.82		
Pan-Shank	5	86.86±5.75		
GluN1	5	93.77±5.92		
GluN2A	5	80.52±13.74		
GluR1	5	88.57±6.79		
GluR2	5	91.55±5.07		
vGluT1	5	93.36±4.96		
PICK1	5	98.62±2.87		
Calvindin	5	104.4±13.49		

Supplementary Fig 3A, B

		N number	Mean ±S.E.M	Anderson-Darling test			Statistics	P-value /Significance
				Omnibus A2	P-value	Pass normality test		
Input Resistance	Ctrl	33	128.6±5.74	0.9880	0.9681	Yes	Mann-Whitney test	0.5788/N.S.
	Nrxn2 KO	37	123.4±7.28	0.9721	0.4671	Yes		
Capacitance	Ctrl	33	193.5±8.24	0.2022	0.8678	Yes	Mann-Whitney test	0.3073/N.S.
	Nrxn2 KO	37	207.4±10.36	0.2740	0.6444	Yes		

Supplementary Fig 3C

		N number /Mice	Mean ±S.E.M	Anderson-Darling test			Statistics	P-value /Significance
				Omnibus A2	P-value	Pass normality test		
EPSC rise time	Ctrl	24/4	2.44±0.07	0.3328	0.4753	Yes	Mann-Whitney test	0.5693/N.S.
	Nrxn2 KO	22/3	2.38±0.07	0.2237	0.7671	Yes		
EPSC decay time	Ctrl	24/4	15.89±0.57	0.9843	0.9852	Yes	Mann-Whitney test	0.0394/*
	Nrxn2 KO	22/3	17.7±0.64	0.1402	0.9600	Yes		

Supplementary Fig 3D

		N number /Mice	Mean ±S.E.M	Anderson-Darling test			Statistics	P-value /Significance
				Omnibus A2	P-value	Pass normality test		
EPSC rise time	Ctrl	13/3	2.74±0.28	0.9043	0.0803	Yes	Mann-Whitney test	0.2040/N.S.
	Nrxn2 KO	17/3	2.37±0.13	0.4813	0.2014	Yes		
EPSC decay time	Ctrl	13/3	21.10±1.49	0.9609	0.6482	Yes	Mann-Whitney test	0.0009/**
	Nrxn2 KO	17/3	15.78±0.52	0.2584	0.6718	Yes		

Supplementary Fig 3E

		N number /Mice	Mean ±S.E.M	Anderson-Darling test			Statistics	P-value /Significance
				Omnibus A2	P-value	Pass normality test		
EPSC rise time	Ctrl	23/3	6.31±0.27	0.3378	0.4566	Yes	Mann-Whitney test	0.0482/N.S.
	Nrxn2 KO	23/3	5.71±0.12	0.8169	0.3676	Yes		
EPSC decay time	Ctrl	24/3	160.8±7.53	0.2969	0.5474	Yes	Mann-Whitney test	<0.0001/***
	Nrxn2 KO	24/3	194.2±6.93	0.2542	0.6893	Yes		

Supplementary Fig. 4a

Supplementary Fig. 4b

Normalized Gene Expression	2^						
(CPM(exons+introns)	Exc. Neuron 1 (CA1/Sbc)	Exc. Neuron 2 (CA3)	Exc. Neuron 3 (CA2)	Exc. Neuron 4 (DG)	GABA Neurons	CR Neurons	Astrocyte 1
Nrxn2	14.94516959	17.25182416	28.97878747	29.7361915	25.42789666	3.838981487	19.79179509
Actb	1491.391908	1154.340462	1916.219036	1164.705403	631.3159514	401.7446297	319.3071349

Normalized Gene Expression							
(CPM(exons+introns)	Astrocyte 2	Oligo. 1	Oligo. 2	Oligo. 3	Oligo. 4	Oligo. 5	Oligo. 6
Nrxn2	283.71798	74.68749078	79.45891009	65.45354217	1	1.477370035	1
Actb	505.1077649	511.8692078	1054.897421	1365.300032	1659.030165	1099.008732	948.3557375

Normalized Gene Expression			
(CPM(exons+introns)	Endothelial	Macrophage 1	Macrophage 2
Nrxn2	1	1	1
Actb	1438.240451	1485.720588	1559.118215

Supplementary Fig. 4c

					ActB Normalized
			Nrxn2	ActB	Nrxn2
21	TEGLU21	Excitatory neurons, hippocampus CA1	4.96	6.38	0.777429467
24	TEGLU23	Excitatory neurons, hippocampus CA3	2.99	6.06	0.49339934
23	TEGLU24	Excitatory neurons, hippocampus CA1	1.95	5.69	0.342706503
26	DGGRC2	Granule neurons, dentate gyrus	0.747	1.49	0.501342282
60	TEINH10	R-LM border Cck interneurons, cortex/hippocampus	2.17	5.03	0.431411531
61	TEINH11	R-LM border Cck interneurons, cortex/hippocampus	5.32	4.59	1.159041394
58	TEINH12	Non-border Cck interneurons, cortex/hippocampus	5.87	5.71	1.028021016
57	TEINH13	Trilaminar cells, hippocampus	3.91	4.04	0.967821782
55	TEINH14	CGE-derived neurogliaform cells Cxcl14+, cortex/hippocampus	1.62	1.95	0.830769231
54	TEINH15	CGE-derived neurogliaform cells, cortex/hippocampus	1.43	1.89	0.756613757
53	TEINH16	Ivy and MGE-derived neurogliaform cells, cortex/hippocampus	2.18	3.03	0.719471947
49	TEINH17	Axo-axonic, cortex/hippocampus	1.52	2.87	0.529616725
50	TEINH18	Basket and bistratified cells, cortex/hippocampus	2.32	3.35	0.692537313
51	TEINH19	Hippocamposeptal projection, cortex/hippocampus	3.17	3.92	0.808673469
56	TEINH20	Inhibitory interneurons, hippocampus	2.73	4.61	0.592190889
52	TEINH21	Sleep-active, long-range projection interneurons, cortex/hippocampus	2.02	2.77	0.729241877
62	TEINH4	Interneuron-selective interneurons, cortex/hippocampus	1.86	1.61	1.155279503
63	TEINH5	Interneuron-selective interneurons, cortex/hippocampus	1.69	1.49	1.134228188
66	TEINH6	Interneuron-selective interneurons, cortex/hippocampus	1.8	1.6	1.125
65	TEINH7	Interneuron-selective interneurons, hippocampus	1.12	5.63	0.198934281
64	TEINH8	Interneuron-selective interneurons, hippocampus	1.02	4.56	0.223684211

59	TEINH9	Non-border Cck interneurons, hippocampus	2.06	4.37	0.471395881
	AVERAGED	GABA Neurons	2.4338889	3.50111111	0.752996278
134	CR	Cajal-Retzius cells, hippocampus	0.474	0.926	0.51187905
231	ACTE1	Telencephalon astrocytes, fibrous	0.834	1.17	0.712820513
232	ACTE2	Telencephalon astrocytes, protoplasmic	0.489	0.593	0.824620573
229	RGDG	Dentate gyrus radial glia-like cells	0.291	0.302	0.963576159
25	DGGRC1	Granule neuroblasts, dentate gyrus	0.768	2.13	0.36056338
34	DGNBL2	Granule neuroblasts, dentate gyrus	0.537	3.24	0.165740741
35	DGNBL1	Granule neuroblasts, dentate gyrus	0.288	3.43	0.083965015
		Avg. DG Neuroblasts	0.531	2.93333333	0.203423045
239	OPC	Oligodendrocytes precursor cells	0.999	1.82	0.548901099
215	COP1	Committed oligodendrocytes cells (COP)	0.357	3.71	0.096226415
218	NFOL1	Newly formed oligodendrocytes (NFOL)	0.084	4.22	0.019905213
219	MFOL2	Myelin forming oligodendrocytes (MFOL)	0.288	2.97	0.096969697
220	MFOL1	Myelin forming oligodendrocytes (MFOL)	0.576	4.11	0.140145985
221	MOL1	Mature oligodendrocytes	0.39	2.9	0.134482759
227	EPEN	Ependymal cells	0.735	4.9	0.15
259	VECC	Vascular endothelial cells, capillary	0.03	6	0.005
260	VECV	Vascular endothelial cells, venous	0.021	3.84	0.00546875
254	VECA	Vascular endothelial cells, arterial	0.384	5.24	0.073282443
		Averaged Endothelial Cells	0.145	5.02666667	0.027917064
255	PER3	Pericytes	0.12	1.28	0.09375
257	PER1	Pericytes	0.09	4.64	0.019396552
258	PER2	Pericytes, possibly mixed with VENC	0.138	10.6	0.013018868

		Averaged Pericyte	0.116	5.50666667	0.04205514
263	MGL3	Microglia, activated	0.024	2.43	0.009876543
264	MGL2	Microglia, activated	0.018	1.94	0.009278351
265	MGL1	Microglia	0.03	1.81	0.016574586
		Averaged Microglia	0.024	2.06	0.011909826

Supplementary Fig 4C, F

		N number /Mice	Mean ±S.E.M	Anderson-Darling test			Statistics	P-value /Significance
				Omnibus A2	P-value	Pass normality test		
AMPAR EPSC rise time	Ctrl	12/4	2.43±0.31	0.7418	0.6435	Yes	Mann-Whitney test	0.9447/N.S.
	Nrxn2 nKO	12/4	2.40±0.25	0.4458	0.2328	Yes		
AMPAR EPSC decay time	Ctrl	12/4	33.12±3.40	0.5028	0.1641	Yes	Mann-Whitney test	0.2317/N.S.
	Nrxn2 nKO	12/4	28.58±1.45	0.6492	0.0677	Yes		
NMDAR EPSC decay time	Ctrl	12/4	227.8±42.7	0.9271	0.3507	Yes	Mann-Whitney test	0.7465/N.S.
	Nrxn2 nKO	12/4	213.6±8.04	0.3542	0.3995	Yes		

Supplementary Fig 5B

		N number /Mice	Mean ±S.E.M	Anderson-Darling test			Statistics	P-value /Significance
				Omnibus A2	P-value	Pass normality test		
vGluT1	Ctrl	18/3	102.3±3.631	0.3442	0.4700	Yes	Mann-Whitney test	0.4354/N.S.
	Nrxn2 nKO	18/3	105.2±3.619	0.1427	0.9635	Yes		
Homer1	Ctrl	18/3	100±7.154	0.9400	0.2898	Yes	Mann-Whitney test	0.1895/N.S.
	Nrxn2 nKO	18/3	107.2±5.743	0.3192	0.5077	Yes		

Supplementary Fig 5C

		N number /Mice	Mean ±S.E.M	Anderson-Darling test			Statistics	P-value /Significance
				Omnibus A2	P-value	Pass normality test		
vGluT1	Ctrl	-/3	106±15.8	0.9643	0.6369	Yes	Mann-Whitney test	>0.9999/N.S.
	Nrxn2 nKO	-/3	107.7±6.367	0.9999	0.9779	Yes		
Homer1	Ctrl	-/3	107±18.43	0.9643	0.6369	Yes	Mann-Whitney test	>0.9999/N.S.
	Nrxn2 nKO	-/3	110.5±11.49	0.8993	0.3831	Yes		

Supplementary Fig 6D

Stimulation intensity		N number /Mice	Mean ±S.E.M	Statistics	Interaction	Stimulation intensity factor	Group factor	P-value /Significance
40	ΔCre	10/4	92.67±23.22	Two-way ANOVA	P<0.0001	P<0.0001	P<0.0001	0.9954/N.S.
	Cre	10/4	64.28±10.60					
50	ΔCre	10/4	216.7±54.54					0.7381/N.S.
	Cre	10/4	160±10.6					
60	ΔCre	10/4	311.3±43.92					0.4718/N.S.
	Cre	10/4	250.8±18.1					
70	ΔCre	10/4	398±75.27					0.9963/N.S.
	Cre	10/4	377.6±30.5					
80	ΔCre	10/4	468.1±73.59					0.9845/N.S.
	Cre	10/4	508.3±44.89					
90	ΔCre	10/4	603.6±54.91					0.9999/N.S.
	Cre	10/4	625.8±54.68					
100	ΔCre	10/4	760.5±76.3					0.9998/N.S.
	Cre	10/4	798.5±65.45					

Supplementary Fig 6E

	N number /Mice	Mean ±S.E.M	Anderson-Darling test			Statistics	P-value /Significance
			Omnibus A2	P-value	Pass normality test		
ΔCre	10/4	7.746±0.6259	0.2562	0.6295	Yes	Mann-Whitney test	0.8633/N.S.
Cre	10/4	7.941±0.7716	0.2773	0.5604	Yes		

Supplementary Fig 6G

	N number /Mice	Mean ±S.E.M	Anderson-Darling test			Statistics	P-value /Significance
			Omnibus A2	P-value	Pass normality test		
ΔCre	10/4	0.3203±0.0219	0.3608	0.3484	Yes	Mann-Whitney test	0.8785/N.S.
Cre	10/4	0.3170±0.02594	0.7314	0.3510	Yes		

Supplementary Fig 6I

	N number /Mice	Mean ±S.E.M	Anderson-Darling test			Statistics	P-value /Significance
			Omnibus A2	P-value	Pass normality test		
ΔCre	10/4	1.314±0.0379	0.3150	0.4810	Yes	Mann-Whitney test	0.8534/N.S.
Cre	10/4	1.319±0.0356	0.3025	0.5115	Yes		

Supplementary Fig 7A

		N number	Mean ±S.E.M	Statistics	Interaction	Time factor	Group factor
1	ΔCre	9	18.8±0.81	Two-way ANOVA	P=0.2079	P<0.0001	P<0.0001
	Cre	10	13.84±1.76				
2	ΔCre	9	24.16±2.48				
	Cre	10	18.56±1.83				
3	ΔCre	9	27.29±2.34				
	Cre	10	20.09±1.83				
4	ΔCre	9	29.17±2.02				
	Cre	10	21.67±1.98				
5	ΔCre	9	31.61±2.30				
	Cre	10	25.45±1.98				
6	ΔCre	9	32.19±2.18				
	Cre	10	26.2±2.19				
7	ΔCre	9	30.41±2.47				
	Cre	10	28.87±2.01				
8	ΔCre	9	32.25±2.40				
	Cre	10	28.36±2.13				
9	ΔCre	9	33.27±2.36				
	Cre	10	28.39±2.26				

Supplementary Fig 7B

		N number	Mean ±S.E.M	Statistics	Interaction	Time factor	Group factor
Baseline	ΔCre	9	40.90±15.32	Two-way ANOVA	P=0.9976	P=0.6647	P=0.5404
	Cre	10	35.79±12.54				
Recall 1	ΔCre	9	63.98±15.96				
	Cre	10	46.84±16.05				
Recall 2	ΔCre	9	52.70±17.05				
	Cre	10	51.44±17.06				
Recall 3	ΔCre	9	47.86±18.08				
	Cre	10	42.92±17.28				

Supplementary Fig 7C

		N number	Mean ±S.E.M	Statistics	Interaction	Time factor	Group factor
Baseline	ΔCre	9	0.35±0.16	Two-way ANOVA	P=0.2858	P<0.0001	P=0.5174
	Cre	10	3.18±3.06				
1	ΔCre	9	12.82±3.23				
	Cre	10	12.50±3.18				
2	ΔCre	9	31.22±5.79				
	Cre	10	29.84±5.69				
3	ΔCre	9	51.39±5.22				
	Cre	10	46.45±5.77				
4	ΔCre	9	60.28±5.60				
	Cre	10	63.89±4.59				
5	ΔCre	9	67.20±8.05				
	Cre	10	73.16±4.49				
6	ΔCre	9	79.43±4.43				
	Cre	10	66.79±5.94				

Supplementary Fig 7D

		N number	Mean ±S.E.M	Anderson-Darling test			Statistics	P-value /Significance
				Omnibus A2	P-value	Pass normality test		
Context	ΔCre	9	37.70±5.62	0.3312	0.4167	Yes	Mann-Whitney test	0.6334/N.S.
	Cre	10	42.36±7.50	0.4799	0.1727	Yes		
Altered context	ΔCre	9	18.97±4.14	0.3921	0.2849	Yes	Mann-Whitney test	0.3548/N.S.
	Cre	10	21.39±3.12	0.2349	0.7081	Yes		
Cued	ΔCre	9	51.49±9.04	0.3749	0.3197	Yes	Mann-Whitney test	0.7225/N.S.
	Cre	10	47.06±8.17	0.4098	0.2680	Yes		

REFERENCES AND NOTES

1. J. R. Sanes, S. L. Zipursky, Synaptic specificity, recognition molecules, and assembly of neural circuits. *Cell* **181**, 536–556 (2020).

2. H. Y. Kim, J. W. Um, J. Ko, Proper synaptic adhesion signaling in the control of neural circuit architecture and brain function. *Prog. Neurobiol.* **200**, 101983 (2021).

3. S. Jang, H. Lee, E. Kim, Synaptic adhesion molecules and excitatory synaptic transmission. *Curr. Opin. Neurobiol.* **45**, 45–50 (2017).

4. A. Ribic, T. Biederer, Emerging roles of synapse organizers in the regulation of critical periods. *Neural Plast.* **2019**, 1538137 (2019).

5. T. C. Südhof, The cell biology of synapse formation. *J. Cell Biol.* **220**, e202103052 (2021).

6. D. D. Krueger, L. P. Tuffy, T. Papadopoulos, N. Brose, The role of neurexins and neuroligins in the formation, maturation, and function of vertebrate synapses. *Curr. Opin. Neurobiol.* **22**, 412–422 (2012).

7. X. Cao, K. Tabuchi, Functions of synapse adhesion molecules neurexin/neuroligins and neurodevelopmental disorders. *Neurosci. Res.* **116**, 3–9 (2017).

8. A. M. Gomez, L. Traunmuller, P. Scheiffele, Neurexins: Molecular codes for shaping neuronal synapses. *Nat. Rev. Neurosci.* **22**, 137–151 (2021).

9. G. Rudenko, Neurexins—Versatile molecular platforms in the synaptic cleft. *Curr. Opin. Struct. Biol.* **54**, 112–121 (2019).

10. T. C. Südhof, Synaptic neurexin complexes: A molecular code for the logic of neural circuits. *Cell* **171**, 745–769 (2017).

11. M. Yuzaki, Two classes of secreted synaptic organizers in the central nervous system. *Annu. Rev. Physiol.* **80**, 243–262 (2018).

12. N. J. Allen, D. A. Lyons, Glia as architects of central nervous system formation and function. *Science* **362**, 181–185 (2018).

13. Y. A. Ushkaryov, A. G. Petrenko, M. Geppert, T. C. Südhof, Neurexins: Synaptic cell surface proteins related to the α-latrotoxin receptor and laminin. *Science* **257**, 50–56 (1992).

14. Y. A. Ushkaryov, Y. Hata, K. Ichtchenko, C. Moomaw, S. Afendis, C. A. Slaughter, T. C. Südhof, Conserved domain structure of β-neurexins. Unusual cleaved signal sequences in receptor-like neuronal cell-surface proteins. *J. Biol. Chem.* **269**, 11987–11992 (1994).

15. B. Ullrich, Y. A. Ushkaryov, T. C. Südhof, Cartography of neurexins: More than 1000 isoforms generated by alternative splicing and expressed in distinct subsets of neurons. *Neuron* **14**, 497–507 (1995).

16. K. Tabuchi, T. C. Südhof, Structure and evolution of neurexin genes: Insight into the mechanism of alternative splicing. *Genomics* **79**, 849–859 (2002).

17. F. H. Sterky, J. H. Trotter, S.-J. Lee, C. V. Recktenwald, X. Du, B. Zhou, P. Zhou, J. Schwenk, B. Fakler, T. C. Südhof, Carbonic anhydrase-related protein CA10 is an evolutionarily conserved pan-neurexin ligand. *Proc. Natl. Acad. Sci. U.S.A.* **114**, E1253-E1262 (2017).

18. E. Rees, J. T. R. Walters, L. Georgieva, A. R. Isles, K. D. Chambert, A. L. Richards, G. Mahoney-Davies, S. E. Legge, J. L. Moran, S. A. McCarroll, M. C. O'Donovan, M. J. Owen, G. Kirov, Analysis of copy number variations at 15 schizophrenia-associated loci. *Br. J. Psychiatry* **204**, 108–114 (2014).

19. T. Bourgeron, From the genetic architecture to synaptic plasticity in autism spectrum disorder. *Nat. Rev. Neurosci.* **16**, 551–563 (2015).

20. M. V. Fuccillo, C. Pak, Copy number variants in neurexin genes: Phenotypes and mechanisms. *Curr. Opin. Genet. Dev.* **68**, 64–70 (2021).

21. E. Kasem, T. Kurihara, K. Tabuchi, Neurexins and neuropsychiatric disorders. *Neurosci. Res.* **127**, 53–60 (2018).

22. Z. Hu, X. Xiao, Z. Zhang, M. Li, Genetic insights and neurobiological implications from NRXN1 in neuropsychiatric disorders. *Mol. Psychiatry* **24**, 1400–1414 (2019).

23. T. C. Südhof, Neuroligins and neurexins link synaptic function to cognitive disease. *Nature* **455**, 903–911 (2008).

24. C. Pak, T. Danko, Y. Zhang, J. Aoto, G. Anderson, S. Maxeiner, F. Yi, M. Wernig, T. C. Südhof, Human neuropsychiatric disease modeling using conditional deletion reveals synaptic transmission defects caused by heterozygous mutations in NRXN1. *Cell Stem Cell* **17**, 316–28 (2015).

25. C. Pak, T. Danko, V. R. Mirabella, J. Wang, Y. Liu, M. Vangipuram, S. Grieder, X. Zhang, T. Ward, Y.-W. A. Huang, K. Jin, P. Dexheimer, E. Bardes, A. Mitelpunkt, J. Ma, M. McLachlan, J. C. Moore, P. Qu, C. Purmann, J. L. Dage, B. J. Swanson, A. E. Urban, B. J. Aronow, Z. P. Pang, D. F. Levinson, M. Wernig, T. C. Südhof, Cross-platform validation of neurotransmitter release impairments in schizophrenia patient-derived NRXN1-mutant neurons. *Proc. Natl. Acad. Sci. U.S.A.* **118**, e2025598118 (2021).

26. Y. Hata, S. Butz, T. C. Südhof, CASK: A novel dlg/PSD95 homolog with an N-terminal calmodulin-dependent protein kinase domain identified by interaction with neurexins. *J. Neurosci.* **16**, 2488–2494 (1996).

27. U. Moog, T. Bierhals, K. Brand, J. Bautsch, S. Biskup, T. Brune, J. Denecke, C. E. de Die-Smulders, C. Evers, M. Hempel, M. Henneke, H. Yntema, B. Menten, J. Pietz, R. Pfundt, J. Schmidtke, D. Steinemann, C. T. Stumpel, L. Van Maldergem, K. Kutsche, Phenotypic and molecular insights into *CASK*-related disorders in males. *Orphanet J. Rare Dis.* **10**, 44 (2015).

28. B. Treutlein, O. Gokce, S. R. Quake, T. C. Südhof, Cartography of neurexin alternative splicing mapped by single-molecule long-read mRNA sequencing. *Proc. Natl. Acad. Sci. U.S.A.* **111**, E1291-E129 (2014).

29. D. Schreiner, T.-M. Nguyen, G. Russo, S. Heber, A. Patrignani, E. Ahrné, P. Scheiffele, Targeted combinatorial alternative splicing generates brain region-specific repertoires of neurexins. *Neuron* **84**, 386–98 (2014).

30. T. Uemura, M. Mishina, The amino-terminal domain of glutamate receptor delta2 triggers presynaptic differentiation. *Biochem. Biophys. Res. Commun.* **377**, 1315–1319 (2008).

31. S. Sugita, F. Saito, J. Tang, J. Satz, K Cambell, T. C. Südhof, A stoichiometric complex of neurexins and dystroglycan in brain. *J. Cell Biol.* **154**, 435–446 (2001).

32. M. Missler, W. Zhang, A. Rohlmann, G. Kattenstroth, R. E. Hammer, K. Gottmann, T. C. Südhof, α-Neurexins couple Ca^{2+} channels to synaptic vesicle exocytosis. *Nature* **423**, 939–948 (2003).

33. W. Zhang, A. Rohlmann, V. Sargsyan, G. Aramuni, R. E. Hammer, T. C. Südhof, M. Missler, Extracellular domains of α-neurexins participate in regulating synaptic transmission by selectively affecting N- and P/Q-type Ca^{2+} channels. *J. Neurosci.* **25**, 4330–4342 (2005).

34. I. Dudanova, K. Tabuchi, A. Rohlmann, T. C. Südhof, M. Missler, Deletion of α-neurexins does not cause a major impairment of axonal pathfinding or synapse formation. *J. Comp. Neurol.* **502**, 261–274 (2007).

35. L. Y. Chen, M. Jiang, B. Zhang, O. Gokce, T. C. Südhof, Conditional deletion of all neurexins defines diversity of essential synaptic organizer functions for neurexins. *Neuron* **94**, 611–625.e4 (2017).

36. F. Luo, A. Sclip, M. Jiang, T. C. Südhof, Neurexins cluster Ca^{2+} channels within the presynaptic active zone. *EMBO J.* **39**, e103208 (2020).

37. F. Luo, A. Sclip, S. Merrill, T. C. Südhof, Neurexins regulate presynaptic GABAB-receptors at central synapses. *Nat. Commun.* **12**, 2380 (2021).

38. M. R. Etherton, C. A. Blaiss, C. M. Powell, T. C. Südhof, Mouse neurexin-1α deletion causes correlated electrophysiological and behavioral changes consistent with cognitive impairments. *Proc. Natl. Acad. Sci. U.S.A.* **106**, 17998–18003 (2009).

39. J. Aoto, C. Foldy, S. M. Ilcus, K. Tabuchi, T. C. Südhof, Distinct circuit-dependent functions of presynaptic neurexin-3 at GABAergic and glutamatergic synapses. *Nat. Neurosci.* **18**, 997–1007 (2015).

40. J. H. Trotter, Z. Dargaei, A. Sclip, S. Essayan-Perez, K. Liakath-Ali, K. Raju, A. Nabet, X. Liu, T. C. Südhof, Compartment-specific neurexin nanodomains orchestrate tripartite synapse assembly. bioRxiv 2020.08.21.262097 [**Preprint**]. 18 May 2021. https://doi.org/10.1101/2020.08.21.262097.

41. J. Aoto, D. C. Martinelli, R. C. Malenka, K. Tabuchi, T. C. Südhof, Presynaptic neurexin-3 alternative splicing trans-synaptically controls postsynaptic AMPA receptor trafficking. *Cell* **154**, 75–88 (2013).

42. J. Dai, J. Aoto, T. C. Südhof, Alternative splicing of presynaptic neurexins differentially controls postsynaptic NMDA and AMPA receptor responses. *Neuron* **102**, 993–1008.e5 (2019).

43. J. Dai, C. Patzke, K. Liakath-Ali, E. Seigneur, T. C. Südhof, GluD1 is a signal transduction device disguised as an ionotropic receptor. *Nature* **595**, 261–265 (2021).

44. G. Born, H. M. Grayton, H. Langhorst, I. Dudanova, A. Rohlmann, B. W. Woodward, D. A. Collier, C. Fernandes, M. Missler, Genetic targeting of NRXN2 in mice unveils role in excitatory cortical synapse function and social behaviors. *Front. Synaptic. Neurosci.* **7**, 3 (2015).

45. J. H. Trotter, J. Hao, S. Maxeiner, T. Tsetsenis, Z. Liu, X. Zhuang, T. C. Südhof, Synaptic neurexin-1 assembles into dynamically regulated active zone nanoclusters. *J. Cell Biol.* **218**, 2677–2698 (2019).

46. A. J. Khalaj, F. H. Sterky, A. Sclip, J. Schwenk, A. T. Brunger, B. Fakler, T. C. Südhof, Deorphanizing FAM19A proteins as pan-neurexin ligands with an unusual biosynthetic binding mechanism. *J. Cell Biol.* **219**, e202004164 (2020).

47. X. Zhan, M. Cao, A. S. Yoo, Z. Zhang, L. Chen, G. R. Crabtree, J. I. Wu, Generation of BAF53b-Cre transgenic mice with pan-neuronal Cre activities. *Genesis* **53**, 440–448 (2015).

48. N. A. Hessler, A. M. Shirke, R. Malinow, The probability of transmitter release at a mammalian central synapse. *Nature* **366**, 569–572 (1993).

49. C. Rosenmund, J. D. Clements, G. L. Westbrook, Nonuniform probability of glutamate release at a hippocampal synapse. *Science* **262**, 754–757 (1993).

50. A. Dani, B. Huang, J. Bergan, C. Dulac, X. Zhuang, Superresolution imaging of chemical synapses in the brain. *Neuron* **68**, 843–856 (2010).

51. A. Attardo, J. E. Fitzgerald, M. J. Schnitzer, Impermanence of dendritic spines in live adult CA1 hippocampus. *Nature* **523**, 592–596 (2015).

52. T. Pfeiffer, S. Poll, S. Bancelin, J. Angibaud, V. K. Inavalli, K. Keppler, M. Mittag, M. Fuhrmann, U. V. Nägerl, Chronic 2P-STED imaging reveals high turnover of dendritic spines in the hippocampus in vivo. *eLife* **7**, e34700 (2018).

53. C. Y. Wang, J. H. Trotter, K. Liakath-Ali, S.-J. Lee, X. Liu, T. C. Südhof, Molecular self-avoidance in synaptic neurexin complexes. *Sci. Advances* **7**, eabk1924 (2021).

54. A. Zeisel, H. Hochgerner, P. Lönnerberg, A. Johnsson, F. Memic, J. van der Zwan, M. Häring, E. Braun, L. E. Borm, G. La Manno, S. Codeluppi, A. Furlan, K. Lee, N. Skene, K. D. Harris, J. Hjerling-Leffler, E. Arenas, P. Ernfors, U. Marklund, S. Linnarsson, Molecular architecture of the mouse nervous system. *Cell* **174**, 999–1014.e22 (2018).

55. X. Xu, W. Morishita, P. S. Buckmaster, Z. P. Pang, R. C. Malenka, T. C. Südhof, Distinct neuronal coding schemes in memory revealed by selective erasure of fast synchronous synaptic transmission. *Neuron* **73**, 990–1001 (2012).

56. X. Zhang, P.-Y. Lin, K. Liakath-Ali, T. C. Südhof, Teneurins assemble into presynaptic nanoclusters that promote synapse formation via postsynaptic non-teneurin ligands. *Nat. Commun.* **13**, 2297 (2022).

57. J. Liang, W. Xu, Y.-T. Hsu, A. X. Yee, L. Chen, T. C. Südhof, Conditional neuroligin-2 knockout in adult medial prefrontal cortex links chronic changes in synaptic inhibition to cognitive impairments. *Mol. Psychiatry* **20**, 850–859 (2015).

58. M. F. Juette, T. J. Gould, M. D. Lessard, M. J. Mlodzianoski, B. S. Nagpure, B. T. Bennett, S. T. Hess, J. Bewersdorf, Three-dimensional sub-100 nm resolution fluorescence microscopy of thick samples. *Nat. Methods* **5**, 527–529 (2008).

59. M. J. Mlodzianoski, M. F. Juette, G. L. Beane, J. Bewersdorf, Experimental characterization of 3D localization techniques for particle-tracking and super-resolution microscopy. *Opt. Express* **17**, 8264–8277 (2009).

60. G. R. Anderson, J. Aoto, K. Tabuchi, C. Földy, J. Covy, A. X. Yee, D. Wu, S.-J. Lee, L. Chen, R. C. Malenka, T. C. Südhof, β-Neurexins control neural circuits by regulating synaptic endocannabinoid signaling. *Cell* **162**, 593–606 (2015).

61. S. R. Guariglia, K. K. Chadman, Water T-maze: A useful assay for determination of repetitive behaviors in mice. *J. Neurosci. Methods* **220**, 24–29 (2013).

62. S. C. Fowler, B. R. Birkestrand, R. Chen, S. J. Moss, E. Vorontsova, G. Wang, T. J. Zarcone, A force-plate actometer for quantitating rodent behaviors: Illustrative data on locomotion, rotation, spatial patterning, stereotypies, and tremor. *J. Neurosci. Methods* **107**, 107–124. (2001).

63. A. Saunders, E. Z. Macosko, A. Wysoker, M. Goldman, F. M. Krienen, H. de Rivera, E. Bien, M. Baum, L. Bortolin, S. Wang, A. Goeva, J. Nemesh, N. Kamitaki, S. Brumbaugh, D.

Kulp, S. A. McCarroll, Molecular diversity and specializations among the cells of the adult mouse brain. *Cell* **174**, 1015–1030.e16 (2018).

64. R. D. Hodge, T. E. Bakken, J. A. Miller, K. A. Smith, E. R. Barkan, L. T. Graybuck, J. L. Close, B. Long, N. Johansen, O. Penn, Z. Yao, J. Eggermont, T. Höllt, B. P. Levi, S. I. Shehata, B. Aevermann, A. Beller, D. Bertagnolli, K. Brouner, T. Casper, C. Cobbs, R. Dalley, N. Dee, S.-L. Ding, R. G. Ellenbogen, O. Fong, E. Garren, J. Goldy, R. P. Gwinn, D. Hirschstein, C. D. Keene, M. Keshk, A. L. Ko, K. Lathia, A. Mahfouz, Z. Maltzer, M. McGraw, T. N. Nguyen, J. Nyhus, J. G. Ojemann, A. Oldre, S. Parry, S. Reynolds, C. Rimorin, N. V. Shapovalova, S. Somasundaram, A. Szafer, E. R. Thomsen, M. Tieu, G. Quon, R. H. Scheuermann, R. Yuste, S. M. Sunkin, B. Lelieveldt, D. Feng, L. Ng, A. Bernard, M. Hawrylycz, J. W. Phillips, B. Tasic, H. Zeng, A. R. Jones, C. Koch, E. S. Lein, Conserved cell types with divergent features in human versus mouse cortex. *Nature* **573**, 61–68 (2019).

65. B. Tasic, Z. Yao, L. T. Graybuck, K. A. Smith, T. N. Nguyen, D. Bertagnolli, J. Goldy, E. Garren, M. N. Economo, S. Viswanathan, O. Penn, T. Bakken, V. Menon, J. Miller, O. Fong, K. E. Hirokawa, K. Lathia, C. Rimorin, M. Tieu, R. Larsen, T. Casper, E. Barkan, M. Kroll, S. Parry, N. V. Shapovalova, D. Hirschstein, J. Pendergraft, H. A. Sullivan, T. K. Kim, A. Szafer, N. Dee, P. Groblewski, I. Wickersham, A. Cetin, J. A. Harris, B. P. Levi, S. M. Sunkin, L. Madisen, T. L. Daigle, L. Looger, A. Bernard, J. Phillips, E. Lein, M. Hawrylycz, K. Svoboda, A. R. Jones, C. Koch, H. Zeng, Shared and distinct transcriptomic cell types across neocortical areas. *Nature* **563**, 72–78 (2018).
